# SREBP1c-CRY1 signalling represses hepatic glucose production by promoting FOXO1 degradation during refeeding

**DOI:** 10.1038/ncomms12180

**Published:** 2016-07-14

**Authors:** Hagoon Jang, Gha Young Lee, Christopher P. Selby, Gung Lee, Yong Geun Jeon, Jae Ho Lee, Kenneth King Yip Cheng, Paul Titchenell, Morris J. Birnbaum, Aimin Xu, Aziz Sancar, Jae Bum Kim

**Affiliations:** 1 School of Biological Sciences, Institute of Molecular Biology and Genetics, Center for Adipose Tissue Remodeling, Seoul National University, Seoul 151-742, Korea; 2 Department of Biochemistry and Biophysics, University of North Carolina School of Medicine, CB # 7260, Chapel Hill, North Carolina 27599-7260, USA; 3 State Key Laboratory of Pharmaceutical Biotechnology and Department of Medicine, The University of Hong Kong, Hong Kong, 999077, China; 4 The Institute for Diabetes, Obesity and Metabolism, Perelman School of Medicine, University of Pennsylvania, Philadelphia, Pennsylvania 19104, USA

## Abstract

SREBP1c is a key lipogenic transcription factor activated by insulin in the postprandial state. Although SREBP1c appears to be involved in suppression of hepatic gluconeogenesis, the molecular mechanism is not thoroughly understood. Here we show that CRY1 is activated by insulin-induced SREBP1c and decreases hepatic gluconeogenesis through FOXO1 degradation, at least, at specific circadian time points. *SREBP1c*^*−/−*^ and *CRY1*^*−/−*^ mice show higher blood glucose than wild-type (WT) mice in pyruvate tolerance tests, accompanied with enhanced expression of *PEPCK* and *G6Pase* genes. CRY1 promotes degradation of nuclear FOXO1 by promoting its binding to the ubiquitin E3 ligase MDM2. Although SREBP1c fails to upregulate CRY1 expression in *db/db* mice, overexpression of CRY1 attenuates hyperglycaemia through reduction of hepatic FOXO1 protein and gluconeogenic gene expression. These data suggest that insulin-activated SREBP1c downregulates gluconeogenesis through CRY1-mediated FOXO1 degradation and that dysregulation of hepatic SREBP1c-CRY1 signalling may contribute to hyperglycaemia in diabetic animals.

## Abstract (web summary)

The clock protein Cry regulates hepatic glucose metabolism. Here the authors show that SREBP1c, activated by insulin signalling after feeding, directly regulates Cry transcription at specific circadian time points, and that Cry represses hepatic glucose production by promoting proteasomal degradation of Foxo1.

## Graphical abstract

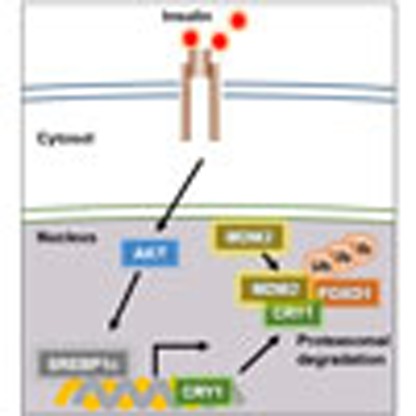

Insulin, which is released from pancreatic β-cells, plays a key role in the maintenance of the whole body energy homoeostasis by actively regulating glucose and lipid metabolism. In the postprandial state, insulin lowers blood glucose by stimulating glucose uptake in adipose tissues and muscles as well as by inhibiting hepatic glucose production^1^,^2^. Moreover, in the liver, insulin stimulates the conversion of excess glucose into glycogen (glycogenesis) and triacylglyceride (lipogenesis) for the long-term energy storage^3^,^4^,^5^.

Suppression of hepatic gluconeogenesis by insulin is an important process to inhibit hyperglycaemia. PEPCK and G6Pase are crucial enzymes that convert pyruvate to glucose, and their gene expression is regulated by several transcription factors such as Forkhead box O1 (FOXO1), cAMP response element-binding protein (CREB), hepatocyte nuclear factor 4 (HNF4), glucocorticoid receptor and peroxisome proliferator-activated receptor gamma coactivator 1-alpha (PGC1α)^6^,^7^,^8^. In the liver, FOXO1 is activated on fasting and gets inactivated by feeding, which is one of the essential mechanisms by which insulin rapidly and efficiently represses hepatic glucose production during postprandial periods^9^,^10^,^11^. After insulin treatment, FOXO1 protein is phosphorylated by AKT and then moves to the cytoplasm, resulting in the decrease of gluconeogenic gene expression^12^. Although the translocation of hepatic FOXO1 from the nucleus to the cytoplasm is a well-defined mechanism mediating a quick decrease in glucose production by insulin, it is largely unknown how insulin endows a sustainable inhibition of hepatic gluconeogenesis throughout the postprandial state.

On the other hand, SREBP1c has been proposed to be involved in the regulation of hepatic glucose metabolism. SREBP1c is a basic-helix-loop-helix-leucine zipper (bHLH-LZ) transcription factor that regulates *de novo* lipogenesis^13^,^14^,^15^,^16^,^17^. Activation of SREBP1c is mediated by AKT and mTORC1 on insulin signalling^18^,^19^. SREBP1c regulates lipogenic pathways by stimulating the expression of target genes such as those encoding fatty acid synthase (*FASN*), stearoyl-CoA desaturase 1 (*SCD1*), and acetyl-coenzyme A carboxylase (*ACC*)^20^,^21^,^22^. In addition, SREBP1c appears to regulate hepatic carbohydrate metabolism. For example, SREBP1c affects the mRNA levels of *PEPCK*, *G6Pase* and *IRS-2* genes and inhibits the interaction between HNF4 and PGC1α to suppress gluconeogenic genes^23^,^24^,^25^,^26^,^27^. Although hepatic SREBP1c has been reported to be upregulated in obese animals, the reason why increased SREBP1c fails to repress hepatic gluconeogenesis is unknown. Thus, understanding the molecular mechanisms by which SREBP1c could modulate gluconeogenesis under physiological and pathological conditions is important.

CRY1 is a member of the mammalian clock genes regulated by transcription–translation feedback loop that also includes CLOCK, BMAL1, PER1, PER2 and CRY2, to modulate rhythmic oscillations. CLOCK and BMAL1 form a heterodimer to activate *PER* and *CRY* genes and then elevated PER and CRY proteins act as transcriptional repressors that decrease the transcriptional activity of CLOCK and BMAL1 (refs 28, 29, 30, 31). The hepatic circadian clock is regulated by food intake and by the expression of hormones such as insulin and glucagon, whereas the suprachiasmatic nucleus circadian clock is controlled by the light–dark cycle^32^. Recently, it has been shown that hepatic circadian clock genes also contribute to glucose homoeostasis. For example, hepatic CRY proteins modulate glucose production by inhibiting the glucagon receptor signalling pathway and binding to glucocorticoid receptor^33^,^34^. In addition, an agonist of CRY proteins has been reported to repress the expression of hepatic gluconeogenic genes, such as *PEPCK* and *G6Pase*^35^. Furthermore, *BMAL1*^*−/−*^ mice exhibited disrupted hepatic glucose homoeostasis^36^. However, the molecular mechanisms by which CRY1 could repress hepatic glucose production during the postprandial state remain to be elucidated.

The fact that SREBP1c downregulates hepatic gluconeogenesis prompted us to investigate novel target genes of SREBP1c by comparing *SREBP1c*^+/+^ and *SREBP1c*^*−/−*^ mice. Here we demonstrate that SREBP1c attenuates hepatic glucose production via the activation of CRY1 at certain circadian time points (particularly at ZT 3), eventually leading to degradation of the FOXO1 protein on insulin signalling. While hepatic FOXO1 is rapidly translocated from the nucleus into the cytoplasm by AKT-mediated phosphorylation triggered by insulin, the SREBP1c-CRY1 signalling pathway durably represses the execution of gluconeogenic genes by decreasing nuclear FOXO1 protein for long-term insulin action. In addition, in the liver of diabetic *db/db* mice, overexpression of CRY1 lowers blood glucose, accompanied with attenuated gluconeogenic gene expression and FOXO1 protein. Altogether, our data suggest that insulin activates the SREBP1c-CRY1 signalling pathway, resulting in FOXO1 degradation mediated by MDM2, which is one of the crucial mechanisms to maintain hepatic glucose homoeostasis.

## Results

### CRY1 is upregulated by feeding and insulin

**Figure 1:**
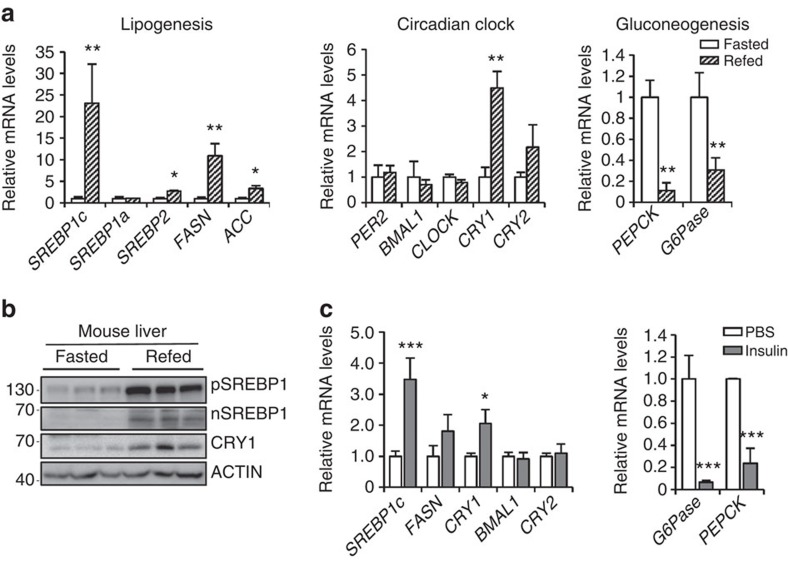
CRY1 is stimulated by feeding and exposure to insulin. (**a**,**b**) *C57BL/6* mice were fasted for 24 h and then refed for 12 h. Both fasted and refed mice were sacrificed at ZT 3. In the liver, levels of the *CRY1* mRNA (**a**) and CRY1 protein (**b**) were determined using qRT-PCR with normalization to *TBP* mRNA levels and western blotting, respectively. pSREBP1, precursor SREBP1; nSREBP1, nuclear SREBP1. Data are represented as mean ±s.d., *N*=3 for each group. *P<0.05, **P<0.01 (Student's *t*-test). (**c**) *CRY1* gene expression was measured in mouse primary hepatocytes following 12 h of insulin exposure using qRT-PCR. The level of the *TBP* mRNA was used for the qRT-PCR normalization. Data are represented as mean±s.d., *N*=3 for each group. *P<0.05, ***P<0.001 (Student's *t*-test). See Supplementary Fig. 13 for original full immunoblot.

In the liver, peripheral circadian clock genes are regulated by various nutritional and hormonal changes^37^. Given that circadian clock genes are closely associated with energy homoeostasis, we sought to investigate which circadian clock genes could affect feeding-dependent hepatic lipid and glucose metabolism. Since the expression of most circadian clock genes oscillates in a time dependent manner, the end points of the fasting and/or refeeding experiment were fixed at ZT 3. On refeeding, the expression of lipogenic genes including *SREBP1c*, *FASN* and *ACC* was upregulated in the liver. In accordance with previous reports^38^,^39^,^40^, the expression of gluconeogenic genes such as *PEPCK* and *G6Pase* was downregulated in the postprandial state (Fig. 1a, Supplementary Fig. 1A). Interestingly, the level of hepatic *CRY1* mRNA was elevated in refed mice (Fig. 1a). Moreover, the expression of the CRY1 protein was markedly increased in the liver of refed mice at ZT 3 (Fig. 1b, Supplementary Fig. 1B). Because CRY1 is one of core circadian clock genes, we investigated the expression of CRY1 gene at ZT 22 and ZT 10, which are the peak and trough time point of CRY1 expression, respectively^31^. On refeeding, the expression of many circadian clock genes including *CRY1, BMAL1, CLOCK, PER1, PER2* and *Reverbs* was significantly changed at ZT 22, but mostly not at ZT 10 (Supplementary Fig. 2). While basal levels of CRY1 expression were differently regulated at ZT 22 and ZT 10, refeeding increased CRY1 mRNA and protein levels at ZT 22, compared with ZT 10. These results imply that CRY1 expression would be modulated by nutritional stimuli at certain ZT time points (Supplementary Fig. 2). These findings prompted us to test whether insulin might elevate hepatic *CRY1* gene expression. In primary hepatocytes, the level of *CRY1* mRNA was increased by insulin, similar to *SREBP1c* mRNA (Fig. 1c). Altogether, these data propose that hepatic CRY1 expression would be upregulated by feeding and insulin.

### SREBP1c regulates *CRY1* gene expression

**Figure 2:**
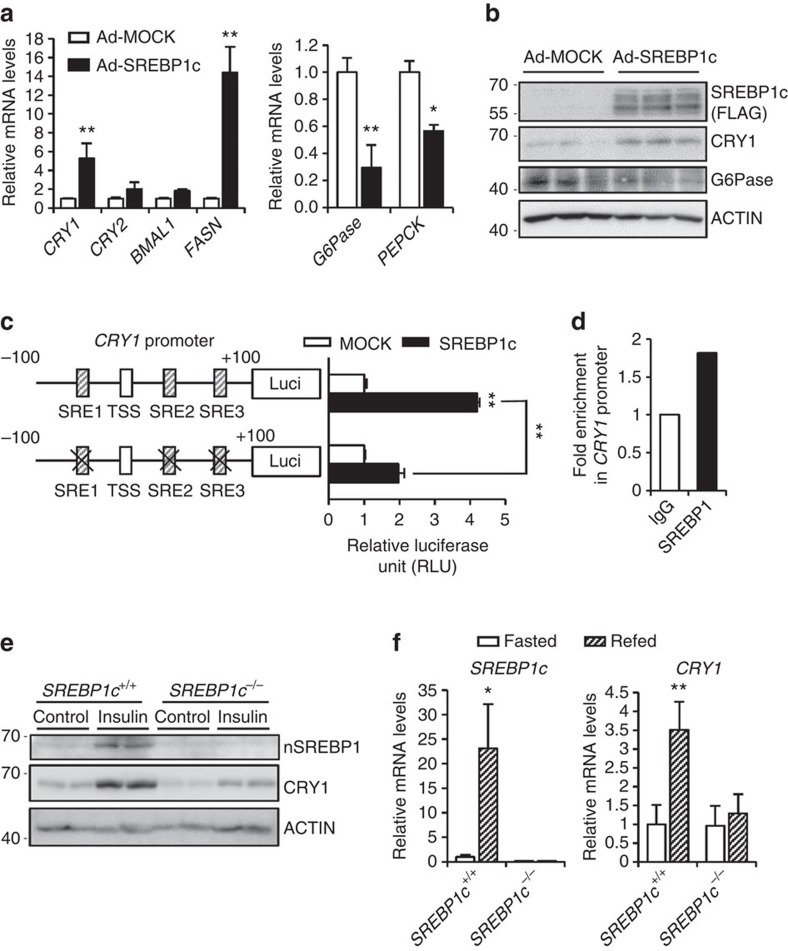
SREBP1c directly activates *CRY1* gene expression. (**a**,**b**) Mouse primary hepatocytes were adenovirally infected with Ad-MOCK or Ad-SREBP1c, as indicated. The levels of the *CRY1* mRNA (**a**) and CRY1 protein (**b**) were determined using qRT-PCR with normalization to *TBP* mRNA levels and western blotting, respectively. Data are represented as mean ±s.d., *N*=3 for each group. *P<0.05, **P<0.01 (Student's *t*-test). (**c**) Luciferase activity of the WT *CRY1* promoter and 3XSRE mutant promoter were measured following co-transfection with expression plasmids encoding either SREBP1c or MOCK in HEK293T cells. Luciferase activity was normalized by β-gal activity. TSS, Transcription Start Site; SRE, Sterol Regulatory Elements. Data are represented as mean ±s.d., *N*=5 for each group. **P<0.01 (Student's *t*-test). (**d**) ChIP assay, performed as described in Methods section, showing *CRY1* promoter occupancy by SREBP1 in H4IIE cells. (**e**) Mouse primary hepatocytes were isolated from *SREBP1c*^*−/−*^ and *SREBP1c*^+/+^ mice. With insulin (10 nM), the levels of SREBP1c and CRY1 protein were determined using western blotting. (**f**) *SREBP1c*^*−/−*^ and *SREBP1c*^+/+^ mice were fasted for 24 h and then refed for 12 h. Both fasted and refed mice were sacrificed at ZT 3. The levels of *SREBP1c* and *CRY1* mRNAs were determined by qRT-PCR and normalized to *TBP* mRNA levels. Data are represented as mean ±s.d., *N*=3–4 for each group. *P<0.05, **P<0.01 (Student's *t*-test). See Supplementary Fig. 13 for original full immunoblot.

To investigate which transcription factors regulate insulin-dependent *CRY1* gene expression, we analysed *CRY1* promoters in several species including monkey, cow, sheep, human, rat and mouse (Supplementary Fig. 3A). In the proximal *CRY1* promoter, there are several sterol regulatory element (SRE) motifs as well as an E-BOX (CANNTG) motif, which is also a target motif for BMAL1 and CLOCK, the core circadian clock proteins (Supplementary Fig. 3A). Both SRE and E-BOX motifs are well-known binding targets of SREBP1c with its dual DNA binding specificity^41^,^42^. To examine whether SREBP1c could regulate *CRY1* gene expression, SREBP1c was overexpressed in mouse primary hepatocytes. As shown in Fig. 2a,b, the levels of hepatic *CRY1* mRNA and CRY1 protein were increased by SREBP1c overexpression (Fig. 2a,b, Supplementary Fig. 4A), implying that SREBP1c may be a key transcription factor that upregulates hepatic *CRY1* gene expression in the postprandial state. Next, the effect of ectopic expression of SREBP1c on the *CRY1* promoter activity was examined. Expression of luciferase from a wild-type *CRY1* promoter was compared with the expression from a promoter with mutated SRE motifs (3XSRE) in HEK293T cells (Fig. 2c). We observed substantial loss of promoter activity with loss of the 3XSRE sequences but not E-BOX motif sequences (Fig. 2c, Supplementary Fig. 3B). In addition, SREBP1c binding to the *CRY1* promoter was confirmed by a ChIP assay (Fig. 2d). Meanwhile, consistent with previous reports^23^,^24^,^25^, hepatic SREBP1c reduced *G6Pase* and *PEPCK* expression (Fig. 2a).

To investigate whether SREBP1c might promote CRY1 gene expression on insulin, primary hepatocytes isolated from *SREBP1c*^*+/+*^ and *SREBP1c*^*−/−*^ mice were challenged with insulin. The level of CRY1 protein in *SREBP1c*^*+/+*^ primary hepatocytes was prominently elevated by insulin whereas that of CRY1 protein in *SREBP1c*^*−/−*^ primary hepatocytes was increased to a lesser extent by insulin (Fig. 2e). Moreover, in contrast to *SREBP1c*^+/+^ mice, refeeding failed to increase hepatic *CRY1* gene expression in *SREBP1c*^*−/−*^ mice (Fig. 2f). These data indicate that SREBP1c is a key factor for the upregulation of hepatic *CRY1* gene expression in the postprandial state.

### SREBP1c-CRY1 pathway inhibits hepatic gluconeogenesis

**Figure 3:**
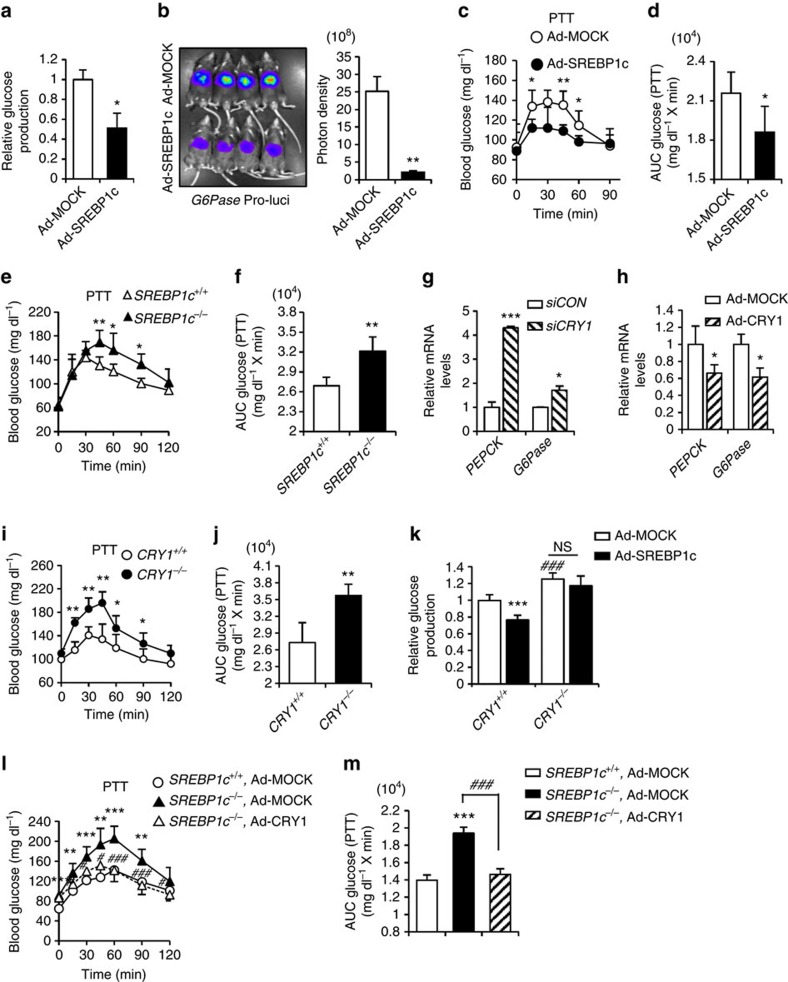
The SREBP1c-CRY1 signalling pathway regulates hepatic glucose production. (**a**) Mouse primary hepatocytes were infected with Ad-MOCK or Ad-SREBP1c. Relative glucose production was measured using a glucose oxidase (GO) kit as described in Methods. Data represent mean ±s.d., *N*=3 for each group. *P<0.05 (Student's *t*-test). (**b**) *C57BL/6* mice were infected with Ad-G6Pase-luc and either Ad-MOCK or Ad-SREBP1c. *G6Pase* promoter activity was measured by optical *in vivo* imaging and photon density. (**c**,**d**) *C57BL/6* mice were infected with Ad-MOCK or Ad-SREBP1c and performed pyruvate tolerance test (**c**). All mice were fasted at ZT 10 and performed PTT at ZT 3. Results were converted to area-under-the curve (AUC) (**d**). Data represent mean ±s.d., *N*=5 for each group. *P<0.05, **P<0.01 (Student's *t*-test). (**e**,**f**) Pyruvate tolerance test (**e**) was performed in *SREBP1c*^*−/−*^ and *SREBP1c*^+/+^mice. All mice were fasted at ZT 10 and performed PTT at ZT 3. Results were converted to AUC (**f**). Data represent mean ±s.d., *N*=5 for each group. *P<0.05, **P<0.01 (Student's *t*-test). (**g**) H4IIE cells were transfected with *siCON* or *siCRY1*. Relative mRNA levels were determined by qRT-PCR and normalized to *cyclophilin* mRNA levels. Data represent mean ±s.d., *N*=3 for each group. *P<0.05, ***P<0.001 (Student's *t*-test). (**h**) Mouse primary hepatocytes were infected with Ad-MOCK or Ad-CRY1. Relative mRNA levels were determined by qRT-PCR and normalized to *TBP* mRNA levels. Data represent mean ±s.d., *N*=3 for each group. *P<0.05 (Student's *t*-test). (**i**,**j**) Pyruvate tolerance test (**i**) was performed in *CRY1*^*−/−*^ and *CRY1*^+/+^ mice. All mice were fasted at ZT 10 and performed PTT at ZT 3. Results were converted to AUC (**j**). Data represent mean ±s.d., *N*=7 for each group. *P<0.05, **P<0.01 (Student's *t*-test). (**k**) Mouse primary hepatocytes isolated from *CRY1*^*+/+*^ and *CRY1*^*−/−*^ mice were infected with Ad-MOCK or Ad-SREBP1c. Relative glucose production was measured using a glucose oxidase (GO) kit. Data represent mean ±s.d., *N*=8 for each group. ***P<0.001 versus Ad-MOCK, ^###^P<0.001 versus *CRY1*^*+/+*^ (Student's *t*-test). (**l**,**m**) Pyruvate tolerance test (**l**) in *SREBP1c*^+/+^ mice injected with Ad-MOCK and in *SREBP1c*^*−/−*^ mice injected with either Ad-MOCK or Ad-CRY1. Results were converted to AUC (**m**). All mice were fasted at ZT 10 and performed PTT at ZT 3. Data represent mean ±s.d., *N*=7–10 for each group. **P<0.01, ***P<0.001 versus *SREBP1c*^+/+^, Ad-MOCK, ^#^*P*<0.05, ^###^*P*<0.001 versus *SREBP1c*^*−/−*^, Ad-MOCK (Student's *t*-test).

Consistent with previous reports^25^,^33^,^34^, SREBP1c overexpression decreased glucose production in mouse primary hepatocytes (Fig. 3a). In addition, the optical *in vivo* imaging analysis revealed that hepatic SREBP1c overexpression remarkably repressed the promoter activity of the *G6Pase* gene *in vivo* (Fig. 3b). Accordingly, the expression of *G6Pase* and *PEPCK* genes was suppressed in the liver of SREBP1c overexpressing mice (Supplementary Fig. 4B). These findings led us to investigate the effect of SREBP1c on blood glucose level *in vivo*. Pyruvate tolerance test revealed that the adenoviral overexpression of SREBP1c significantly decreased the level of blood glucose following pyruvate injection (Fig. 3c,d). Also, *SREBP1c*^*−/−*^ mice showed higher blood glucose than *SREBP1c*^+/+^ mice (Fig. 3e,f). Thus, these results suggest that hepatic SREBP1c would suppress gluconeogenesis, potentially by modulating gluconeogenic gene expression.

To examine whether *CRY1*, a novel target gene of SREBP1c, might modulate hepatic gluconeogenic gene expression, we suppressed *CRY1* expression via small interfering RNA (siRNA) in rat hepatoma H4IIE cells. Downregulation of CRY1 increased the expression of *G6Pase* and *PEPCK* genes (Fig. 3g, Supplementary Fig. 5A), which are crucial for hepatic gluconeogenesis. On the contrary, hepatic CRY1 overexpression decreased the expression of *G6Pase* and *PEPCK* genes in mouse primary hepatocytes (Fig. 3h, Supplementary Fig. 5B). To confirm these observations, we measured pyruvate-induced blood glucose level from *CRY1*^+/+^ and *CRY1*^*−/−*^ mice. As shown in Fig. 3i,j, *CRY1*^*−/−*^ mice exhibited higher blood glucose level than *CRY1*^+/+^ mice. To test whether CRY1 would be a key mediator of the inhibition of hepatic gluconeogenesis by SREBP1c, glucose production assays were performed in primary hepatocytes from *CRY1*^+/+^ and *CRY1*^*−/−*^ mice. As indicated in Fig. 3k and Supplementary Fig. 5C, SREBP1c overexpression attenuated glucose production in *CRY1*^*+/+*^ primary hepatocytes while SREBP1c failed to suppress glucose production in *CRY1*^*−/−*^ primary hepatocytes. To establish whether the SREBP1c-CRY1 signalling pathway could indeed repress hepatic glucose production *in vivo*, CRY1 was adenovirally overexpressed in the liver of *SREBP1c*^*−/−*^ mice. While *SREBP1c*^*−/−*^ mice showed higher blood glucose level than *SREBP1c*^+/+^ mice during the pyruvate tolerance test, *SREBP1c*^*−/−*^ mice with CRY1 overexpression decreased blood glucose level (Fig. 3l,m, Supplementary Fig. 6A,B). These data strongly indicate that the SREBP1c-CRY1 signalling pathway could inhibit hepatic gluconeogenesis *in vivo*.

### CRY1 regulates FOXO1 protein levels

**Figure 4:**
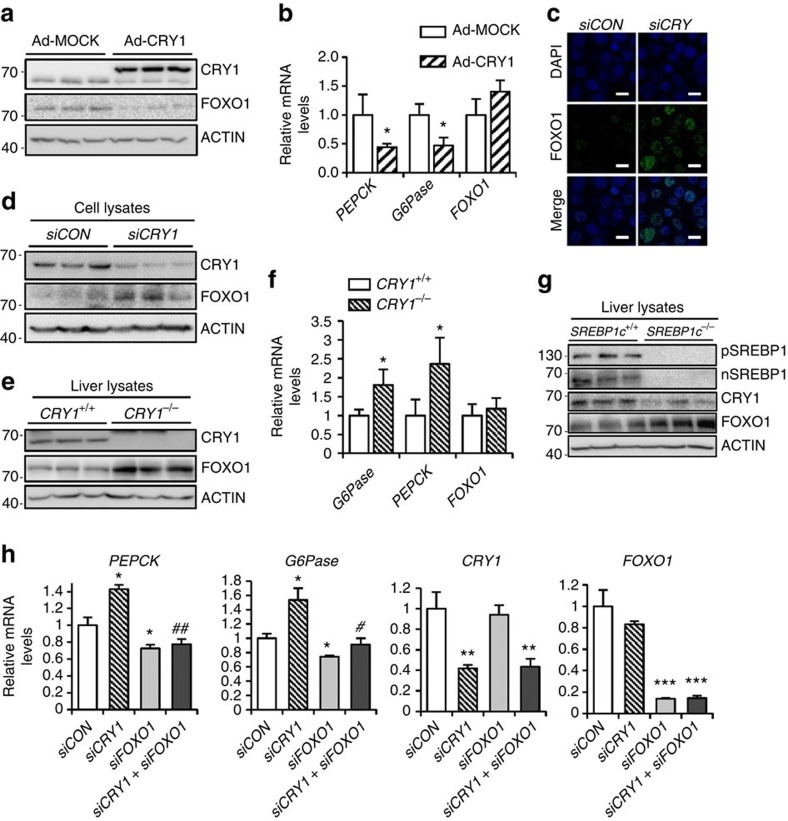
CRY1 regulates FOXO1 protein level. (**a**,**b**) Mouse primary hepatocytes were adenovirally infected with Ad-MOCK or Ad-CRY1. The expression profiles of FOXO1 were analysed at the protein level (**a**) using western blotting and at the mRNA level (**b**) using qRT-PCR. Data are represented as mean ±s.d., *N*=4 for each group. *P<0.05 (Student's *t*-test). (**c**,**d**) H4IIE cells were transfected with *siCON* or *siCRY1*. Immunocytochemical analysis (**c**) of endogenous FOXO1. DAPI, 4', 6-diamidino-2-phenylindole. Scale bars, 10 μm. Endogenous FOXO1 and CRY1 protein levels were analysed using western blotting (**d**). (**e**,**f**) The expression patterns of FOXO1 protein in the liver of *CRY1*^+/+^ and *CRY1*^*−/−*^ mice were analysed by western blotting (**e**) and qRT-PCR (**f**). Relative mRNA levels were determined using qRT-PCR and normalized to the levels of the *TBP* mRNA. Data are represented as mean ±s.d., *N*=3 for each group. *P<0.05, (Student's *t*-test). (**g**) Expression of CRY1 and FOXO1 proteins in the liver of *SREBP1c*^+/+^ and *SREBP1c*^*−/−*^ mice was analysed by western blotting. (**h**) H4IIE cells were co-transfected with *siCRY1* and/or *siFOXO1*. Relative mRNA levels were determined using qRT-PCR and normalized to the *cyclophilin* mRNA level. Data are represented as mean ±s.d., *N*=3 for each group. ^#^*P*<0.05, ^##^*P*<0.01 versus *siCRY1*, *P<0.05, **P<0.01, ***P<0.001 versus *siCON* (Student's *t*-test). See Supplementary Fig. 13 for original full immunoblot.

To decipher the underlying mechanism(s) by which insulin-induced CRY1 could repress hepatic gluconeogenesis, we focused on FOXO1, as its regulatory effects on insulin signalling and gluconeogenesis are well established. In mouse primary hepatocytes, the level of FOXO1 protein was decreased by CRY1 overexpression (Fig. 4a) while *FOXO1* mRNA levels were not altered (Fig. 4b). These data indicated that CRY1 might modulate the level of FOXO1 protein, probably, independent of *FOXO1* mRNA. Furthermore, the level of FOXO1 protein was enhanced in *CRY1* suppressed hepatocytes (Fig. 4c,d). In accordance with these *in vitro* data, the level of FOXO1 protein was higher in the liver of *CRY1*^*−/−*^ mice than in the liver of *CRY1*^+/+^ mice, whereas the levels of *FOXO1* mRNA were not different between *CRY1*^+/+^ and *CRY1*^*−/−*^ mice (Fig. 4e,f). Compared with *SREBP1c*^*+/+*^ mice, the levels of FOXO1 protein and *G6Pase* and *PEPCK* mRNAs were augmented in the liver of *SREBP1c*^*−/−*^ mice whereas that of CRY1 protein was reduced (Fig. 4g, Supplementary Fig. 7). To verify that CRY1 could inhibit hepatic gluconeogenesis via FOXO1, the effects of CRY1 and/or FOXO1 suppression on gluconeogenic gene expression were examined. Increased expression of *G6Pase* and *PEPCK* genes by *CRY1* suppression was abolished when *FOXO1* gene was downregulated by siRNA (Fig. 4h), implying that FOXO1 might be a downstream factor of CRY1 to regulate gluconeogenesis. These *in vivo* and *in vitro* data imply that CRY1 would alleviate hepatic gluconeogenesis through FOXO1.

### FOXO1 protein is decreased by insulin-activated CRY1

**Figure 5:**
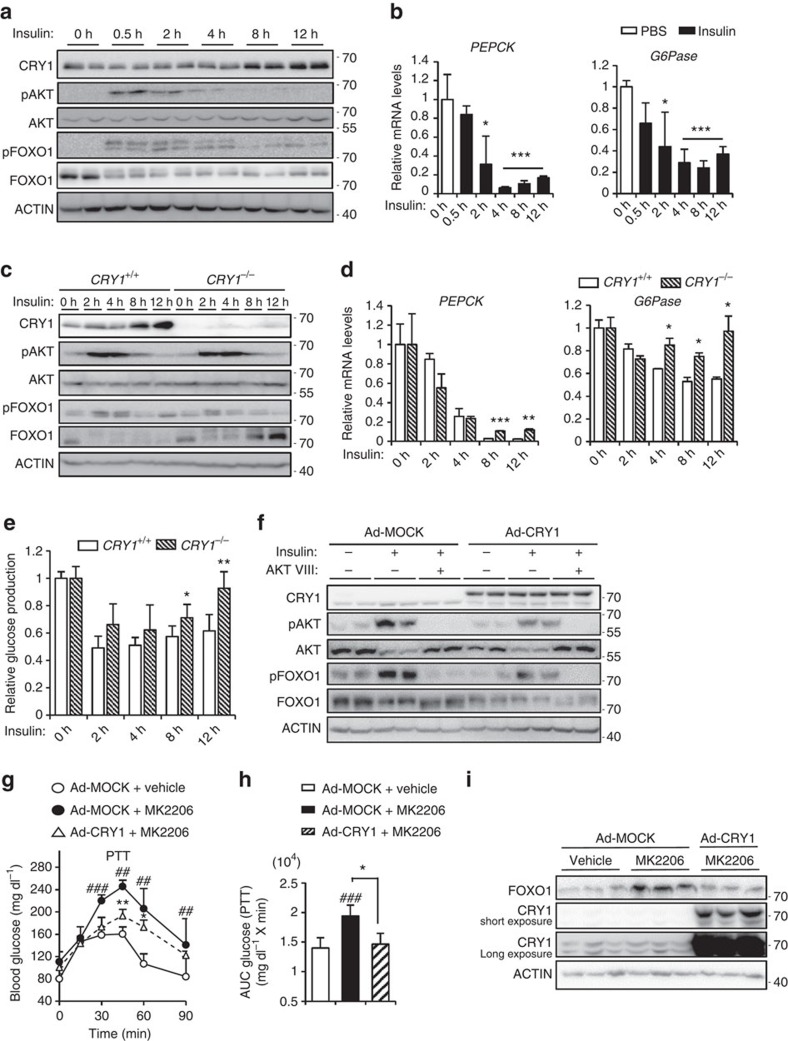
Long-term insulin treatment stimulates CRY1 expression and inhibits hepatic gluconeogenesis. (**a**,**b**) Mouse primary hepatocytes were treated with 10 nM insulin for different periods. Protein levels (**a**) were determined with western blotting. The mRNA levels (**b**) were analysed by qRT-PCR. Data are represented as mean ±s.d., *N*=3 for each group. *P<0.05, ***P<0.001 (Student's *t*-test). (**c**–**e**) Mouse primary hepatocytes isolated from *CRY1*^*+/+*^ and *CRY1*^*−/−*^ mice were treated with 10 nM insulin for different periods. Protein levels (**c**) were analysed by western blotting, and relative mRNA levels (**d**) were determined by qRT-PCR and normalized to the *TBP* mRNA level. Data are represented as mean ±s.d., *N*=3 for each group. *P<0.05, **P<0.01, ***P<0.001 versus *CRY1*^*+/+*^ control (Student's *t*-test). Relative glucose production (**e**) was measured using a glucose oxidase (GO) kit. Data are represented as mean ±s.d., *N*=5 for each group. *P<0.05, **P<0.01, versus *CRY1*^*+/+*^ control (Student's *t*-test). (**f**) Mouse primary hepatocytes were infected with Ad-MOCK and Ad-CRY1, and then treated with insulin (10 nM) or insulin (10 nM) and AKTVIII (5 μM) for 12 h. Protein levels were determined with western blotting. (**g**–**i**) *C57BL/6* mice were infected with Ad-MOCK or Ad-CRY1 and subjected to the pyruvate tolerance test (**g**) with or without the AKT inhibitor MK2206. MK2206 (30 mg kg^−1^) was given by oral gavage 10 min before the pyruvate tolerance test. All mice were fasted at ZT 10 and performed PTT at ZT 3. Results were converted to AUC values (**h**). After the pyruvate tolerance test, hepatic protein levels (**i**) were analysed by western blotting. Data are represented as mean ±s.d., *N*=5–7 for each group. ^##^*P*<0.01, ^###^*P*<0.001 versus Ad-MOCK+vehicle control, *P<0.05, **P<0.01, versus Ad-MOCK+MK2206 control (Student's *t*-test). See Supplementary Fig. 13 for original full immunoblot.

FOXO1 translocation from the nucleus to the cytoplasm by AKT is a well-known mechanism by which insulin acutely inhibits hepatic glucose production^43^. As insulin upregulates CRY1 that, in turn, downregulates the FOXO1 protein (Figs 1c and 4a), we investigated the time course of these events by examining the expression profiles of FOXO1 and CRY1 proteins in insulin-treated primary hepatocytes. As shown in Fig. 5a, phosphorylation of AKT and FOXO1 was clearly induced in cells treated with insulin for a relatively short periods (0–4 h). Consistent with previous report^44^, the level of FOXO1 protein was decreased by short-term insulin treatment (0∼4 h), which would be mediated by FOXO1 ubiquitination and degradation in cytosol. Furthermore, the phosphorylation levels of AKT and FOXO1 were gradually and substantially decreased by a long-term incubation (8–12 h) with insulin. It appears that FOXO1 translocation from the nucleus to the cytoplasm by AKT might be more pronounced after a short exposure (0–4 h) to insulin rather than following a long-term insulin treatment (8–12 h). Intriguingly, in hepatocytes treated with insulin for long periods (8–12 h), the level of the CRY1 protein was markedly increased, while that of the total FOXO1 protein was continuously decreased, implying that the amount of the CRY1 protein appears to be inversely related to the total quantity of the FOXO1 protein. Accordingly, the expression of *PEPCK* and *G6Pase* genes was downregulated after either a long-term (8–12 h) or a short-term (0–4 h) insulin treatment (Fig. 5b). These data suggest that sustainable reduction of the FOXO1 protein might be involved in the suppression of hepatic gluconeogenesis for long-term insulin action.

Next, we explored whether CRY1 could indeed modulate the FOXO1 protein in insulin-treated hepatocytes. To address this, we have tested *CRY1*^*+/+*^ and *CRY1*^*−/−*^ primary hepatocytes. As shown in Fig. 5c, the level of FOXO1 protein was decreased in insulin-treated *CRY1*^*+/+*^ primary hepatocytes while that of FOXO1 protein was not continuously suppressed in insulin-treated *CRY1*^*−/−*^ primary hepatocytes. In the late phase of insulin action, decreased levels of *PEPCK* and *G6Pase* mRNA were slightly but substantially increased in *CRY1*^*−/−*^ primary hepatocytes (Fig. 5d). Moreover, insulin failed to suppress glucose production in long-term (8–12 h) insulin-treated *CRY1*^*−/−*^ primary hepatocytes. (Fig. 5e). Altogether, these data indicate that CRY1 could repress the expression of hepatic gluconeogenic genes with reduced FOXO1 protein for the long-term insulin action (8–12 h).

To test whether enhanced CRY1 could suppress hepatic gluconeogenesis even in the absence of a short-term insulin action (0∼4 h), we employed AKT inhibitors. In primary hepatocytes, insulin increased phosphorylation levels of both AKT and FOXO1, while a co-treatment with the AKT inhibitor AKTVIII blocked phosphorylation of both proteins, as expected (Fig. 5f). However, in CRY1-overexpressing hepatocytes, the total FOXO1 level was decreased in insulin and/or the AKT inhibitor treated cells, implying that CRY1 could downregulate FOXO1 protein independent of FOXO1 phosphorylation (Fig. 5f). To confirm this observation *in vivo*, we tested another AKT inhibitor, MK2206, in mice. As shown in Fig. 5g,h, administration of MK2206 significantly increased blood glucose level on pyruvate challenge; however, adenoviral CRY1 overexpression in mice significantly attenuated blood glucose level even in the presence of MK2206. It is noteworthy that the level of FOXO1 protein was greatly augmented by MK2206, whereas CRY1 elevation suppressed FOXO1 protein expression *in vivo* (Fig. 5i). Taken together, these data clearly indicate that CRY1-dependent FOXO1 reduction may contribute to the suppression of hepatic gluconeogenesis independent of AKT activation.

### CRY1 stimulates proteasomal degradation of FOXO1

**Figure 6:**
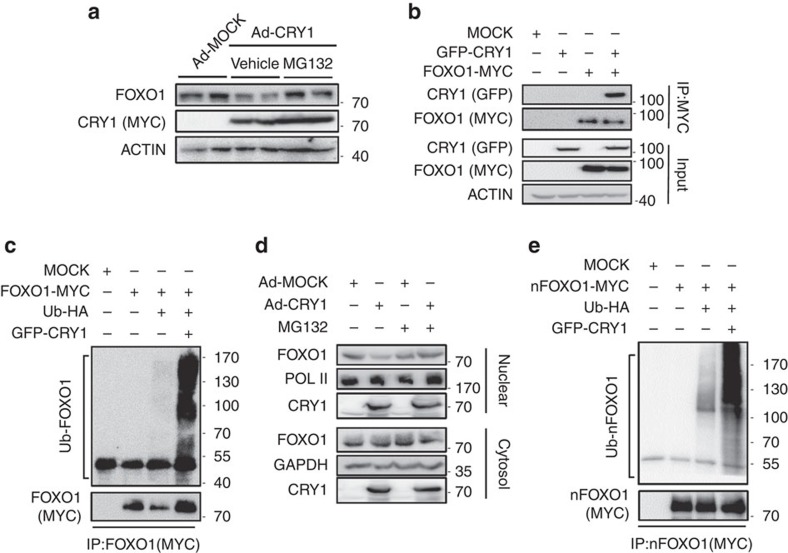
CRY1 accelerates ubiquitin-mediated FOXO1 degradation. (**a**) Mouse primary hepatocytes were adenovirally infected with Ad-MOCK or Ad-CRY1. The cells were treated with 20 μM MG132 or vehicle for 4 h. Total cell lysates were analysed by western blotting with indicated antibodies. (**b**) HEK293T cells were transfected with GFP-CRY1 and/or FOXO1-MYC expression vectors. Co-immunoprecipitation with an anti-MYC antibody and western blotting were performed with the indicated antibodies. IP, immunoprecipitation. (**c**) COS-1 cells were co-transfected with plasmids encoding FOXO1-MYC, GFP-CRY1, and Ubiquitin-HA. After transfection, the cells were treated with MG132 (20 μM) for 6 h and then the cell lysates were subjected to immunoprecipitation with an anti-MYC antibody followed by western blotting with indicated antibodies. IP, immunoprecipitation. (**d**) Mouse primary hepatocytes were infected with Ad-MOCK or Ad-CRY1. After infection, the cells were treated with MG132 (20 μM) for 4 h. Nuclear and cytosolic fractions were isolated and analysed by western blotting with indicated antibodies. (**e**) COS-1 cells were co-transfected with plasmids encoding nFOXO1-MYC, GFP-CRY1, and Ubiquitin-HA. After transfection, the cells were challenged with MG132 (20 μM) for 6 h. The cell lysates were subjected to immunoprecipitation with an anti-MYC antibody. IP, immunoprecipitation. See Supplementary Fig. 13 for original full immunoblot.

Since CRY1 overexpression decreased the level of the FOXO1 protein, but not the *FOXO1* mRNA, we investigated whether the downregulation of the FOXO1 protein might proceed via proteasomal degradation. As shown in Fig. 6a, the reduction of the FOXO1 protein by CRY1 overexpression was alleviated by MG132 treatment, indicating that the regulation of FOXO1 protein by CRY1 may be, at least in part, dependent on proteasomal degradation. When we tested physical interaction between FOXO1 and CRY1 proteins, co-immunoprecipitation assays revealed that CRY1 could associate with FOXO1 protein (Fig. 6b). Then, we examined whether CRY1 might induce FOXO1 degradation via the ubiquitination-proteasome pathway. As shown in Fig. 6c, CRY1 overexpression dramatically promoted FOXO1 poly-ubiquitination, implying that CRY1 could potentiate FOXO1 degradation, probably, through protein–protein interactions.

To further explore the subcellular location of FOXO1 degradation induced by CRY1, the levels of the nuclear and cytosolic FOXO1 protein were investigated. As shown in Fig. 6d, the nuclear fraction of the FOXO1 protein was decreased by CRY1 overexpression whereas incubation with MG132 blocked this decrease. At the same time, the levels of cytosolic FOXO1 were not altered on CRY1 overexpression, irrespective of the presence of MG132. Consistent with these results, poly-ubiquitination of the nuclear form of the FOXO1 mutant protein (nFOXO1-MYC) was greatly augmented by CRY1 (Fig. 6e). Therefore, it is plausible that the degradation of the FOXO1 protein via poly-ubiquitination is stimulated by CRY1 in the nucleus.

### CRY1 is involved in MDM2-mediated FOXO1 degradation

**Figure 7:**
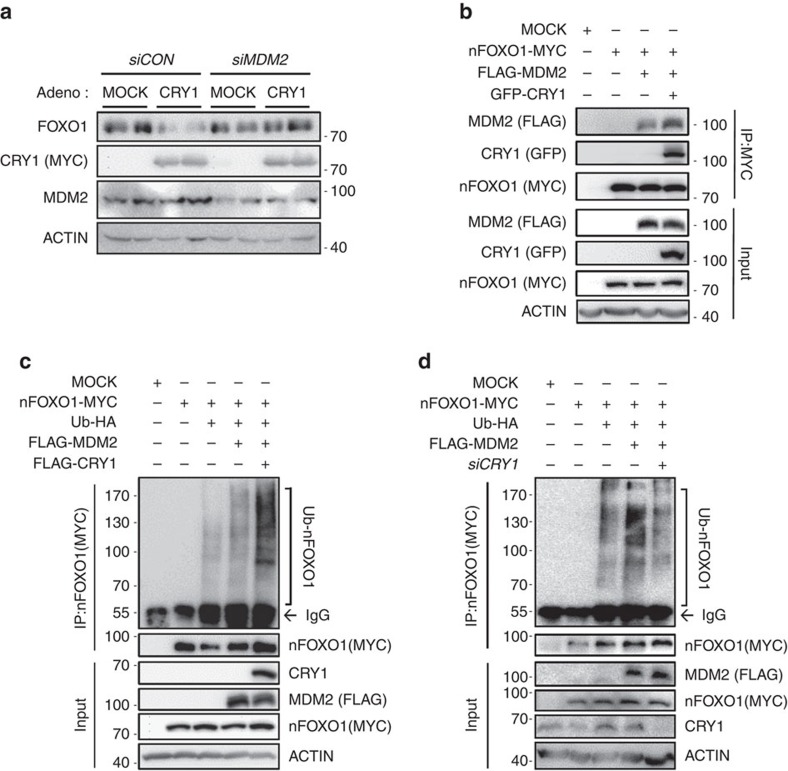
CRY1 is involved in MDM2-mediated FOXO1 ubiquitination. (**a**) Mouse primary hepatocytes were infected with Ad-MOCK or Ad-CRY1 and/or *siCON* or *siMDM2*. Total cell lysates were analysed by western blotting with indicated antibodies. (**b**) HEK293T cells were transfected with FLAG-MDM2, nFOXO1-MYC, and GFP-CRY1 expression vectors. Total cell lysates were subjected to co-immunoprecipitation with an anti-MYC antibody followed by western blotting with indicated antibodies. IP, immunoprecipitation. (**c**) COS-1 cells were co-transfected with plasmids encoding nFOXO1-MYC, FLAG-MDM2, FLAG-CRY1, and Ubiquitin-HA. After transfection, the cells were challenged with MG132 (20 μM) for 6 h. Cell lysates were subjected to immunoprecipitation with an anti-MYC antibody. IP, immunoprecipitation (**d**) COS-1 cells were co-transfected with plasmids encoding nFOXO1-MYC, FLAG-MDM2, Ubiquitin-HA, and *siCRY1*. Cells were treated with MG132 (20 μM) for 6 h. Cell lysates were subjected to immunoprecipitation with an anti-MYC antibody. IP, immunoprecipitation. See Supplementary Fig. 13 for original full immunoblot.

Among several ubiquitin E3 ligases of the FOXO1 protein^45^,^46^, we found that the MDM2 ubiquitin E3 ligase appeared to be involved in the CRY1-mediated FOXO1 degradation. As shown in Fig. 7a, MDM2 suppression markedly rescued the level of the FOXO1 protein in CRY1-overexpressing cells, implying that MDM2 may participate in the CRY1-dependent FOXO1 reduction. To study the role of CRY1 in MDM2-mediated FOXO1 degradation, we tested whether CRY1 might regulate the subcellular localization of MDM2. Wild-type CRY1 and cytosolic CRY1 (ΔNLS-CRY1) did not change the subcellular location of the nuclear MDM2 (Supplementary Fig. 8). Instead, we revealed that CRY1 potentiates the association between FOXO1 and MDM2 (Fig. 7b).

In another experiment, we explored whether CRY1 could modulate MDM2-mediated FOXO1 degradation. As shown in Fig. 7c,d, CRY1 overexpression promoted MDM2-mediated poly-ubiquitination of the nuclear form of FOXO1 protein (Fig. 7c), whereas CRY1 suppression attenuated FOXO1 poly-ubiquitination by MDM2 (Fig. 7d). These data indicate that CRY1 would participate in MDM2-induced FOXO1 degradation and repress FOXO1-mediated hepatic glucose production.

### CRY1 mitigates hyperglycaemia in diabetic mouse models

**Figure 8:**
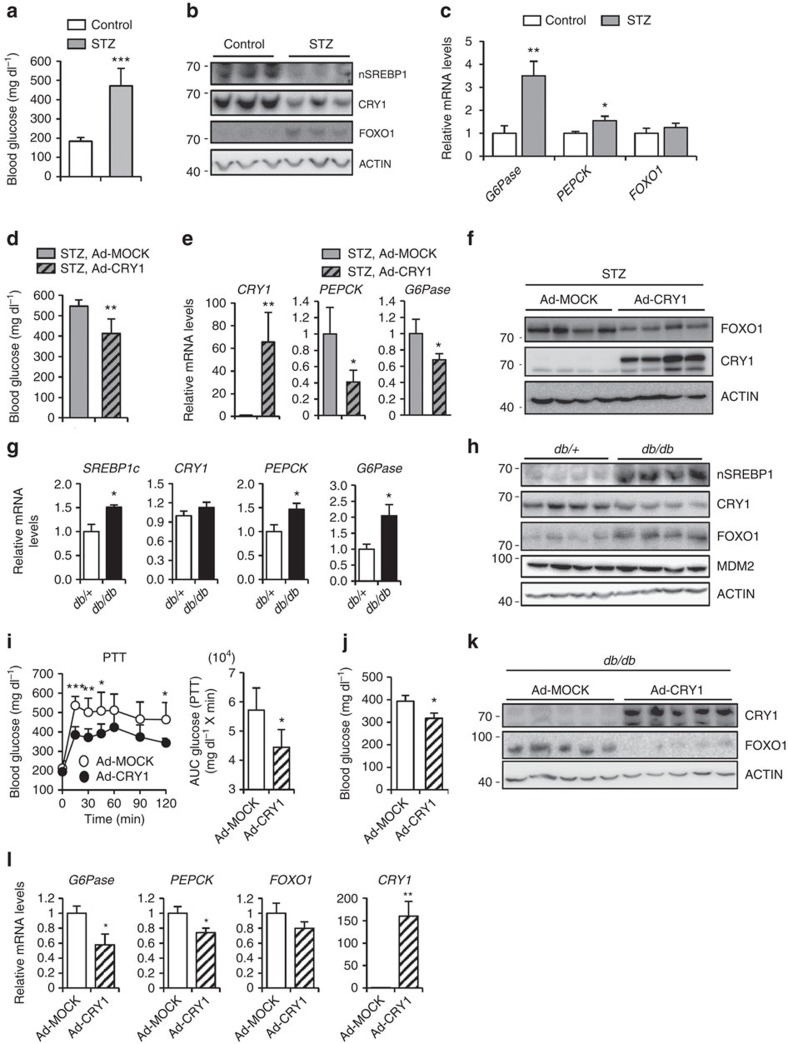
CRY1 alleviates gluconeogenesis in diabetic mouse models. (**a**–**c**) Eight-week-old male mice were injected STZ (150 mg kg^−1^) and sacrificed in ZT 3 after 1 week later. Blood glucose levels (**a**) were measured in ad libitum at ZT 3. After all of the mice were sacrificed at ZT 3, hepatic protein levels (**b**) were analysed by western blotting. The mRNA levels (**c**) were determined by qRT-PCR analyses and normalized to the *TBP* mRNA level. Data represent mean ±s.d., *N*=3 for each group. *P<0.05, **P<0.01, ***P<0.001 (Student's *t*-test). (**d**–**f**) STZ-injected mice were infected with adenovirus encoding MOCK or CRY1 (adenoviral dose of 2 × 10^9^ viral particles per mouse). The blood glucose levels (**d**) were measured in ad libitum at ZT 3. After all of the mice were sacrificed at ZT 3, the mRNA levels (**e**) were determined by qRT-PCR analyses and normalized to the *TBP* mRNA level. Hepatic protein levels (**f**) were analysed by western blotting. Data represent mean ±s.d., *N*=4 for each group. *P<0.05, **P<0.01 (Student's *t*-test). (**g**,**h**) Ten-week-old male *db/+* and *db/db* mice were sacrificed in ad libitum at ZT 3. The relative mRNA levels of various hepatic genes (**g**) were determined by qRT-PCR analyses and normalized to the *TBP* mRNA level. Data represent mean ±s.d., *N*=4 for each group. *P<0.05 (Student's *t*-test). Protein levels (**h**) were determined with western blotting. (**i**–**l**) Ten-week-old male *db/db* mice were infected through the tail vein with adenovirus encoding MOCK or CRY1 (adenoviral dose of 2 × 10^10^ viral particles per mouse) and subjected to the pyruvate tolerance test (**i**). All mice were fasted at ZT 10 and performed PTT at ZT 3. Results were converted to AUC. The blood glucose levels (**j**) were measured in ad libitum at ZT 3. After all of the mice were sacrificed at ZT 3, hepatic protein levels (**k**) were analysed by western blotting. The mRNA levels (**l**) were determined by qRT-PCR analyses and normalized to the *TBP* mRNA level. Data represent mean ±s.d., *N*=5 for each group. *P<0.05, **P<0.01, ***P<0.001 (Student's *t*-test). See Supplementary Fig. 13 for original full immunoblot.

Streptozotocin (STZ)-treated insulin-deficient mice elevated blood glucose as well as gluconeogenic programs including FOXO1 protein levels, accompanied with increased expression of *PEPCK* and *G6Pase* genes (Fig. 8a–c). Since the SREBP1c-CRY1 axis was upregulated by insulin (Figs 1c, 2e), we overexpressed CRY1 in the liver of STZ-treated insulin-deficient mice. As shown in Fig. 8d, CRY1 overexpression diminished blood glucose levels in STZ-treated mice. In addition, CRY1 reduced not only the expression of *PEPCK* and *G6Pase* genes but also the level of FOXO1 protein (Fig. 8e,f). These data suggest that CRY1 would be one of important factors to suppress hepatic glucose production on insulin.

Intriguingly, in the liver of obese animals such as *db/db* and diet-induced obesity mice, SREBP1c level is elevated while hepatic gluconeogenesis is not repressed^47^,^48^,^49^,^50^. To explore which process(es) might be dysregulated in the regulation of hepatic gluconeogenesis, we have examined several mRNA and protein levels for SREBP1c-CRY1 axis and gluconeogenic genes in diabetic animals. Similar to previous reports^47^,^48^,^49^,^50^, the mRNA levels of *SREBP1c* and gluconeogenic genes were elevated in *db/db* mice (Fig. 8g). However, hepatic CRY1 protein was substantially decreased and the level of MDM2 protein was not significantly changed in *db/db* mice (Fig. 8h). Similarly, diet-induced obesity mice exhibited elevated expression of SREBP1c and gluconeogenic genes whereas CRY1 was not activated (Supplementary Fig. 9A,B). To test the idea that dysregulated CRY1 protein might mediate hyperglycaemia with enhanced FOXO1 protein in diabetic animals, CRY1 was adenovirally overexpressed in the liver of *db/db* mice. As shown in Fig. 8i, the levels of pyruvate-induced blood glucose were decreased in CRY1-overexpressing *db/db* mice. Moreover, the levels of feeding blood glucose were diminished by CRY1 overexpression (Fig. 8j). In addition, ectopic CRY1 expression reduced the levels of FOXO1 protein as well as gluconeogenic gene expression in *db/db* mice (Fig. 8k,l). These results indicate that CRY1 could ameliorate hyperglycaemia by repressing the level of FOXO1 protein in *db/db* mice.

## Discussion

As a major anabolic hormone, insulin stimulates lipogenesis and represses gluconeogenesis in the liver. Following insulin exposure, lipogenesis is upregulated by SREBP1c, and the expression of SREBP1c target genes such as *FASN*, *SCD* and *ELOVL6* is induced^20^,^51^,^52^. In contrast, insulin blocks hepatic gluconeogenesis through AKT-mediated phosphorylation of FOXO1 and PGC1α^53^,^54^, both are major regulators of gluconeogenic genes including *PEPCK* and *G6Pase*. Here we propose that the SREBP1c-CRY1 signalling pathway plays an important role to inhibit hepatic gluconeogenesis under anabolic state. Our data from hepatic gluconeogenic gene expression, *in vitro* glucose output assays, time kinetics of insulin signalling cascades, and pyruvate tolerance test, which reflects both hepatic glucose output and peripheral glucose disposal, have consistently suggested the idea that maintenance of SREBP1c-induced CRY1 is crucial to prevent unnecessary hepatic gluconeogenesis during insulin action. In this regard, it has been reported that single-nucleotide polymorphisms of *SREBP1c* and *CRY1* genes are associated with hyperglycaemia in human^55^,^56^.

Similar to previous reports^47^,^48^,^50^, the expression of SREBP1c and gluconeogenic genes was increased in the liver of diabetic *db/db* mice (Fig. 8g,h). Unexpectedly, the level of hepatic CRY1 protein was downregulated in *db/db* mice. Moreover, in the liver of *db/db* mice, diurnal expression of CRY1 protein level was overall reduced whereas FOXO1 protein levels were elevated compared to *db/+* mice (Supplementary Fig. 10A,B). In this work, we have demonstrated that ectopic overexpression of CRY1 in *db/db* mice alleviated hepatic gluconeogenesis by reducing FOXO1 protein (Fig. 8i–l). Also, we have shown that hepatic CRY1 could attenuate the level of blood glucose by decreasing FOXO1 protein, independent of AKT activity (Fig. 5f–i). Although it remains to be elucidated how elevated SREBP1c fails to increase CRY1 in the liver of *db/db* mice, it is very likely that increased FOXO1 protein results from reduced hepatic CRY1 protein in *db/db* mice.

It has been reported that CRY1 seems to suppress hepatic glucose production by interfering with glucocorticoid receptor signalling and the glucagon signalling pathway^33^,^34^,^35^. CRY1 interacts with glucocorticoid receptor^34^ and the α-subunit of the glucagon receptor^33^, which are involved in the regulation of gluconeogenesis. Nonetheless, specific roles of CRY1 during the nutrient-rich state after exposure to insulin have not been clearly elucidated. To test whether CRY1 might repress gluconeogenesis by inhibiting glucagon signalling and/or glucocorticoid receptor-dependent pathway, CRY1-overexpressing primary hepatocytes were treated with forskolin, db-cAMP or dexamethasone to mimic the conditions for glucagon or glucocorticoid stimulation. In primary hepatocytes, CRY1 partially repressed gluconeogenic gene expression in the presence of forskolin, db-cAMP or dexamethasone (Supplementary Fig. 11A–C), implying that in addition to the glucagon and glucocorticoid signalling pathways, there may be another signalling cascade regulated by CRY1, which could suppress hepatic glucose production. Thus, CRY1 appears to be involved in multiple regulatory pathways that control hepatic gluconeogenesis in response to various hormones.

**Figure 9:**
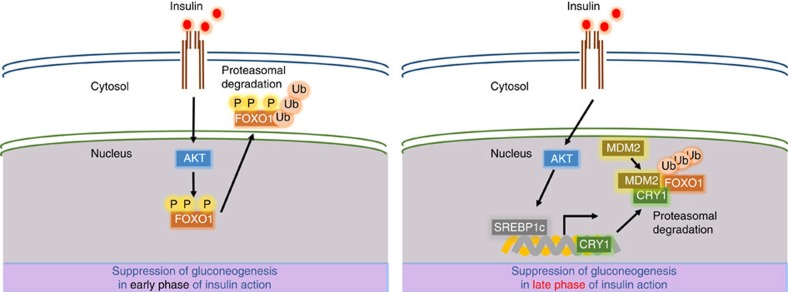
SREBP1c-CRY1 axis suppresses hepatic gluconeogenesis by promoting FOXO1 degradation. For long-term insulin action, SREBP1c-CRY1 axis suppresses hepatic gluconeogenesis through nuclear FOXO1 degradation. During anabolic state, increased CRY1 accelerates MDM2-mediated FOXO1 degradation and thereby insulin sustainably represses hepatic glucose production.

Activation of FOXO1-mediated gluconeogenesis is inhibited by insulin. AKT, a key downstream molecule of the insulin-activated signalling, phosphorylates FOXO1, which then is translocated from the nucleus to the cytoplasm through its association with the 14-3-3 protein^57^. In primary hepatocytes, FOXO1 phosphorylation was rapidly increased by insulin. However, hepatic gluconeogenic programing is persistently and efficiently suppressed regardless of the decreased FOXO1 phosphorylation at the late stage of insulin action. Intriguingly, hepatic CRY1 expression was enhanced at relatively late periods of insulin action (Fig. 5a). Furthermore, in primary hepatocytes, a long-term insulin treatment downregulated FOXO1 expression, while CRY1 deficiency rescued FOXO1 protein levels as well as gluconeogenic gene expression (Fig. 5c,d). It is of interest to note that CRY1-overexpressing mice showed a decrease of blood glucose level as well as of FOXO1 protein when AKT activity was pharmacologically repressed with AKT inhibitor MK2206 (Fig. 5g–i). Collectively, our *in vitro* and *in vivo* data suggest that the CRY1-dependent FOXO1 degradation would be one of the crucial mechanisms in attenuating hepatic gluconeogenesis for the long-term insulin action. Therefore, these observations prompted us to propose that the AKT-mediated FOXO1 phosphorylation provides an acute response during early insulin response, whereas SREBP1c-mediated CRY1 regulation would be a more durable process leading to the repression of futile hepatic gluconeogenesis throughout the anabolic state (Fig. 9).

As one of the key players for circadian rhythmic modulation, CRY1 expression is affected by various transcription factors and epigenetic regulation. Although our *in vivo* and *in vitro* data suggested that CRY1 expression was promoted by insulin and SREBP1c, *CRY1* mRNA expression was not elevated by refeeding at ZT 10, which is the trough point of CRY1 oscillation (Supplementary Fig. 2B). These data propose that increased SREBP1c might not be sufficient to activate CRY1 at ZT 10. Conversely, the levels of CRY1 mRNA and proteins were robustly increased by refeeding at ZT 22, which is the peak point of its oscillation, implying that SREBP1c would augment CRY1 expression, probably, accompanied with other circadian regulatory factors. Nonetheless, it remains to be elucidated whether CRY1 as well as other circadian clock genes may contribute to modulate hepatic gluconeogenesis at ZT 22.

On the other hand, we asked the question whether SREBP1c deficiency would change hepatic circadian clock gene oscillation. As shown in Supplementary Fig. 12, hepatic circadian clock gene oscillations were not significantly different in *SREBP1c*^+/+^ and *SREBP1c*^*−/−*^ mice. Of course, we cannot exclude the possibility that SREBP1c-induced CRY1 might contribute to minor roles for hepatic circadian oscillation in *SREBP1c*^*−/−*^ mice because it has been reported that *CRY1*^*−/−*^ mice exhibit fewer changes in circadian oscillations, compared with *CRY1*^*−/−*^*CRY2*^*−/−*^ double-mutant mice^58^. Furthermore, it is also possible that remaining SREBP1a and/or SREBP2 activity in *SREBP1c*^*−/−*^ mice might maintain intact circadian clock gene oscillations and this homoeostatic regulation needs to be addressed in future studies^21^,^59^. Nonetheless, hepatic *CRY1* gene expression is clearly upregulated by feeding/insulin-mediated SREBP1c.

Consistent with previous reports^31^, hepatic CRY1 was increased at night time (ZT 12–24) when feeding behaviour dominantly occurs in nocturnal animals (Supplementary Fig. 10A). Although *SREBP1c*^*−/−*^ mice did not show any significant difference of circadian clock oscillation (Supplementary Fig. 12), SREBP1c expression was also increased at night time (ZT 12–24; Supplementary Fig. 10). Unlike the expression profiles of SREBP1c and CRY1, the level of FOXO1 protein appeared to be reduced at night time, implying that circadian oscillations of SREBP1c and CRY1 may contribute to repress diurnal glucose production by the regulation of FOXO1.

As SREBP1c could simultaneously regulate both gluconeogenesis and lipogenesis, it is plausible to speculate that hepatic SREBP1c would effectively coordinate the anabolic pathways by upregulating fatty acid metabolism and downregulating glucose metabolism on insulin signalling with different target genes. Our study is the first report to reveal the role of SREBP1c in CRY1 activation, which would be crucial in the regulation of hepatic glucose metabolism in the anabolic state. On the basis of the circadian oscillatory gene expression profile in *SREBP1c*^*−/−*^ mice, it appears that SREBP1c does not actively govern the hepatic circadian clock. Rather, increased expression of hepatic *CRY1* during the postprandial state is primarily regulated, in a clock-gated manner, by insulin-activated SREBP1c, which eventually leads to the suppression of glucose production via FOXO1 degradation (Fig. 9). Although the roles of hepatic CRY1 in energy metabolism need to be investigated further, our data provide an important clue to understand the molecular mechanisms that link hepatic SREBP1c and glucose homoeostasis in physiological and pathological conditions.

## Methods

### Animals

*C57BL/6* male mice were purchased from SAMTACO (Seoul, South Korea) and housed in colony cages. *db/+* and *db/db* male mice were obtained from Central Lab (Seoul, Korea). *SREBP1c*^*−/−*^ mice were generously provided from Dr J. Horton at the University of Texas Southwestern Medical Center and bred in isolated cages. All animals were maintained under 12 h light/12 h dark cycle in a pathogen-free animal facility. Following dissection, mouse tissue specimens were immediately stored at −80 °C until further analysis. All experiments with mice were approved by the Seoul National University Institutional Animal Care and Use Committee (SNUIACUC) and the Institutional Animal Care and Use Committee at the University of North Carolina.

### *In vivo* imaging system

Ten-week-old *C57BL/6* male mice were injected with adenoviruses encoding green fluorescent protein (GFP) (Ad-MOCK), SREBP1c (Ad-SREBP1c) and G6Pase luciferase (Ad-G6Pase-luc) through the tail vein. After 5 days, adenovirus-infected mice were injected intraperitoneally with 100 mg kg^−1^ sterile firefly D-luciferin. After 5 min, mice were anaesthetised and imaged using an IVIS 100 imaging system (Xenogen, Alameda, CA, USA) as described^60^.

### Pyruvate tolerance tests

For the pyruvate tolerance test, mice were fasted for 16 h and then injected intraperitoneally with pyruvate (2 g kg^−1^ body weight for mice). Blood glucose levels were measured in tail vein blood samples at the indicated time points by using a Free-Style blood glucose metre (TheraSense, Sweden).

### Materials and plasmids

MG132 was purchased from Calbiochem (San Diego, CA, USA). MK2206 was purchased from Selleckchem (S1078). AKTVIII was purchased from Santa Cruz Biotechnology (sc-202048). Antibodies against MYC (Cell Signalling, 2276, 1:1,000 dilution), HA (Cell Signalling, 3724, 1:1,000 dilution), FOXO1 (Cell Signalling, 2880, 1:1,000 dilution), phosphor-FOXO1-Ser256 (Cell Signalling, 9461, 1:1,000 dilution), AKT (Cell Signalling, 9272, 1:1,000 dilution), and phosphor-AKT-Ser473 (Cell Signalling, 9271, 1:1,000 dilution), FLAG (Sigma-Aldrich, F3165, 1:1,000 dilution), ACTIN (Sigma-Aldrich, A5316, 1:2,000 dilution), G6Pase (Santa Cruz Biotechnology, sc-33839, 1:500 dilution), POLII (Santa Cruz Biotechnology, sc-899, 1:1,000 dilution), GFP (Santa Cruz Biotechnology, sc-9996, 1:1,000 dilution), GAPDH (LabFrontier, Co., LF-PA0018, 1:1,000 dilution), SREBP1 (BD Bioscience, 557036, 1:1,000 dilution), MDM2 (Abcam, ab16895, 1:1,000 dilution) and CRY1 (lab made antibody from Dr Aziz Sancar^31^, 1:200 dilution) were used. GFP-CRY1 was cloned into the pEGFP-N1 vector and FLAG-MDM2 was cloned into pCMV-3 FLAG. Mouse CRY1 promoter was cloned into the pGL3-basic vector.

### Cell-based ubiquitination assays

COS-1 cells (ATCC, CRL1650) were transfected with plasmids encoding FOXO1 WT-MYC, nFOXO1 (ADA)-MYC, GFP-CRY1 (or FLAG-CRY1), FLAG-MDM2 and Ubiquitin-HA in the presence of 20 μM MG132 for 4 h. Total cell lysates were prepared using the TGN buffer. FOXO1 WT-MYC and nFOXO1 (ADA)-MYC were immunoprecipitated with an anti-MYC antibody, and after washing in the TGN buffer, proteins were separated by SDS–PAGE followed by western blotting analyses with an anti-HA antibody.

### ChIP assays

Cross-linking and chromatin immunoprecipitation assays with H4IIE cells (ATCC, CRL1548) were performed as described previously^61^. Extracted proteins from total cell lysates were immunoprecipitated with anti-SREBP1 (BD Bioscience) or IgG (Santa Cruz) for 2 h. Precipitated DNA fragments were analysed by qRT-PCR using primer sets that encompassed the proximal (−100 to +100 base pairs) region of the rat *CRY1* promoter and negative control (+9670 to +9890 base pairs) region. The sequences of ChIP assay primers were as follows: sense, 5′-GTCCGAGCCAGCGTAGTAAA-3′, antisense, 5′-GGATAGCGCGGGCTAGAG-3′; negative control primer sense, 5′-CCAGCCACTTTGCTGAAGTT-3′ and antisense, 5′-CTAGACAAGGCTGCCCACTC-3′.

### Preparation of recombinant adenovirus

The adenovirus plasmid was constructed as previously described^62^. Rat *SREBP1c* and mouse *CRY1* cDNAs were incorporated into the AdTrack-CMV shuttle vector and a recombinant vector was generated using the Ad-Easy adenoviral vector system. Adenoviruses were amplified in HEK293A cells and isolated by caesium chloride density centrifugation. The GFP was co-expressed from an independent promoter with inserted cDNA. For *in vivo* experiments, mice were injected with 5 × 10^9^ p.f.u. of adenovirus (*db/db* mice, 2 × 10^10^ p.f.u.) in 200 μl PBS through the tail vein. Empty virus expressing only the gene for GFP served as the control (MOCK).

### Mouse primary hepatocytes cultures

Mouse primary hepatocytes were isolated from 10-week-old male mice using collagenase perfusion method^63^ and plated with medium 199 (Invitrogen, Carlsbad, CA, USA). After 6 h of attachment, cells were infected with adenovirus or transfected with siRNA. For adenoviral infection, isolated hepatocytes were incubated for 4 h with adenovirus at 10 PFU per cell with the serum-free medium, which was subsequently replaced by the 10% FBS-containing M199 medium.

### Cell lysis and immunoprecipitation

After washing in cold PBS, cells were treated either with the TGN buffer (50 mM Tris-HCl, pH 7.5, 150 mM NaCl, 1% Tween-20 and 0.3% NP-40) or the SDS lysis buffer (200 mM Tris-HCl, pH 6.8, 10% glycerol and 4% SDS) supplemented with 0.1% protease inhibitor cocktail (Roche, Basel, Switzerland). Total cell lysates were obtained by centrifugation at 12,000 r.p.m. for 15 min at 4 °C, and 1–1.5 mg of lysates was used for immunoprecipitation. The lysates were incubated with primary antibodies for 2 h at 4 °C, followed by 1 h of further incubation with 50% slurry of protein A sepharose presaturated with the lysis buffer. After washing three times with the lysis buffer, the immunoprecipitated proteins were recovered from the beads by boiling for 10 min in the sample buffer and analysed by SDS–PAGE and immunoblotting.

### Transient transfection and luciferase assays

HEK293T cells (ATCC, CRL3216) were transiently transfected with various DNA plasmids using the calcium-phosphate method described previously^64^. After incubation for 36 h, transfected cells were collected with the lysis buffer (25 mM Tris-phosphate pH 7.8, 10% glycerol, 2 mM EDTA, 2 mM DTT and 1% Triton X-100) and the activities of luciferase and β-galactosidase were measured according to the manufacturer's protocol (Promega, Madison, WI, USA). The relative luciferase activity was normalized to β-galactosidase activity in each sample.

### RNA preparation and quantitative reverse transcriptase–PCR analyses

RNA was prepared as previously described^65^. Briefly, total RNA was isolated using the TRIzol reagent (Invitrogen). Subsequently, equal amounts of RNA were subjected to cDNA synthesis using the RevertAid M-MuLV reverse transcriptase (Fermentas, Canada). The relative amount of mRNA was evaluated by using a CFX real-time quantitative PCR detection system (Bio-Rad Laboratories, Hercules, CA, USA) and calculated following normalization to the level of *TBP* or *cyclophilin* mRNA. The primer sequences used for the real-time quantitative PCR analyses are described in the Supplementary Table 1.

### siRNA transfection

siRNA duplexes for *CRY1*, *MDM2,* and *FOXO1* were purchased from the Bioneer, Inc. (Daejeon, South Korea). Primary hepatocytes were transiently transfected with the Lipofectamine RNAi MAX reagent (Invitrogen) according to the manufacturer's protocol. The sequence information for siRNA is described in the Supplementary Table 2.

### Glucose production assays

Glucose production by mouse primary hepatocytes was measured according to the manufacturer's protocol using a glucose oxidase assay (Sigma-Aldrich, St Louis, MO, USA). Briefly, the cells were incubated for 6 h at 37 °C and 5% CO_2_ in the Krebs-Ringer buffer (115 mM NaCl, 5.9 mM KCl, 1.2 mM MgCl_2_, 1.2 mM NaH_2_PO_4_, 2.5 mM CaCl_2_, 25 mM NaHCO_3_, 10 mM lactate and 2 mM pyruvate, pH 7.4). The glucose production assays were performed in triplicate.

### Statistical analysis

Sample sizes were chosen based on pilot experiments that ensured adequate statistical power with similar variances. Statistical significance was assessed by the Student's *t*-test and are presented as the mean±s.d. determined from at least three independent experiments. Values of *P*<0.05 were considered to indicate statistically significant differences. All *n* values defined in the legends refer to biological replicates unless otherwise indicated. If technical failures such as failure of intraperitoneal injection occurred, those samples were excluded from the final analysis. The experiments were not randomized. The investigators were not blinded to allocation during experiments and outcome assessment.

### Data availability

The data that support the findings of this study are available from the corresponding author on reasonable request.

## Additional information

**How to cite this article**: Jang, H. *et al.* SREBP1c-CRY1 signalling represses hepatic glucose production by promoting FOXO1 degradation during refeeding. *Nat. Commun.* 7:12180 doi: 10.1038/ncomms12180 (2016).

## Supplementary Material

Supplementary Information Supplementary Figures 1-13 and Supplementary Tables 1-2
