# Supplementary material for: SREBP1c-CRY1 signalling represses hepatic glucose production by promoting FOXO1 degradation during refeeding
Source: Nat Commun. 2016 Jul 14;7:12180. doi: 10.1038/ncomms12180 (PMC4947181; doi:10.1038/ncomms12180)
Supplement: Supplementary Information — Supplementary Figures 1-13 and Supplementary Tables 1-2 [file ncomms12180-s1.pdf]

Supplementary Figure 1

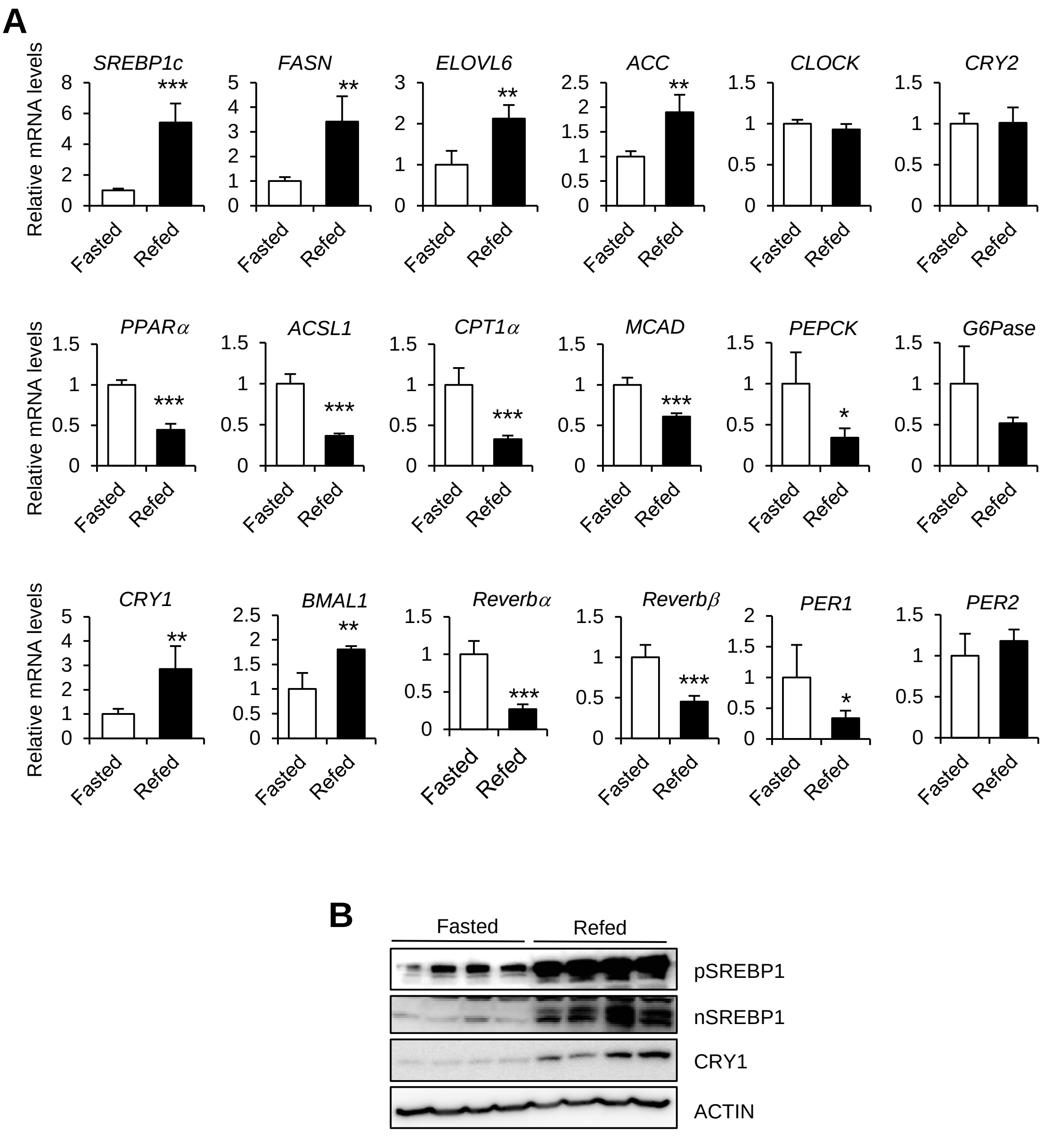

Supplementary Figure 1. CRY1 is increased by feeding

(A and B) *C57BL/6* mice were fasted for 12 hours and then refed for 12 hours. Both fasted and refed mice were sacrificed at ZT 3.In the liver, levels of the *CRY1* mRNA (A) and CRY1 protein (B) were determined using qRT-PCR with normalization to *TBP* mRNA levels and western blotting, respectively. Data are represented as mean ±SD, *N*=4 for each group. *$P<0.05$, **$P<0.01$ (Student's *t*-test).

Supplementary Figure 2

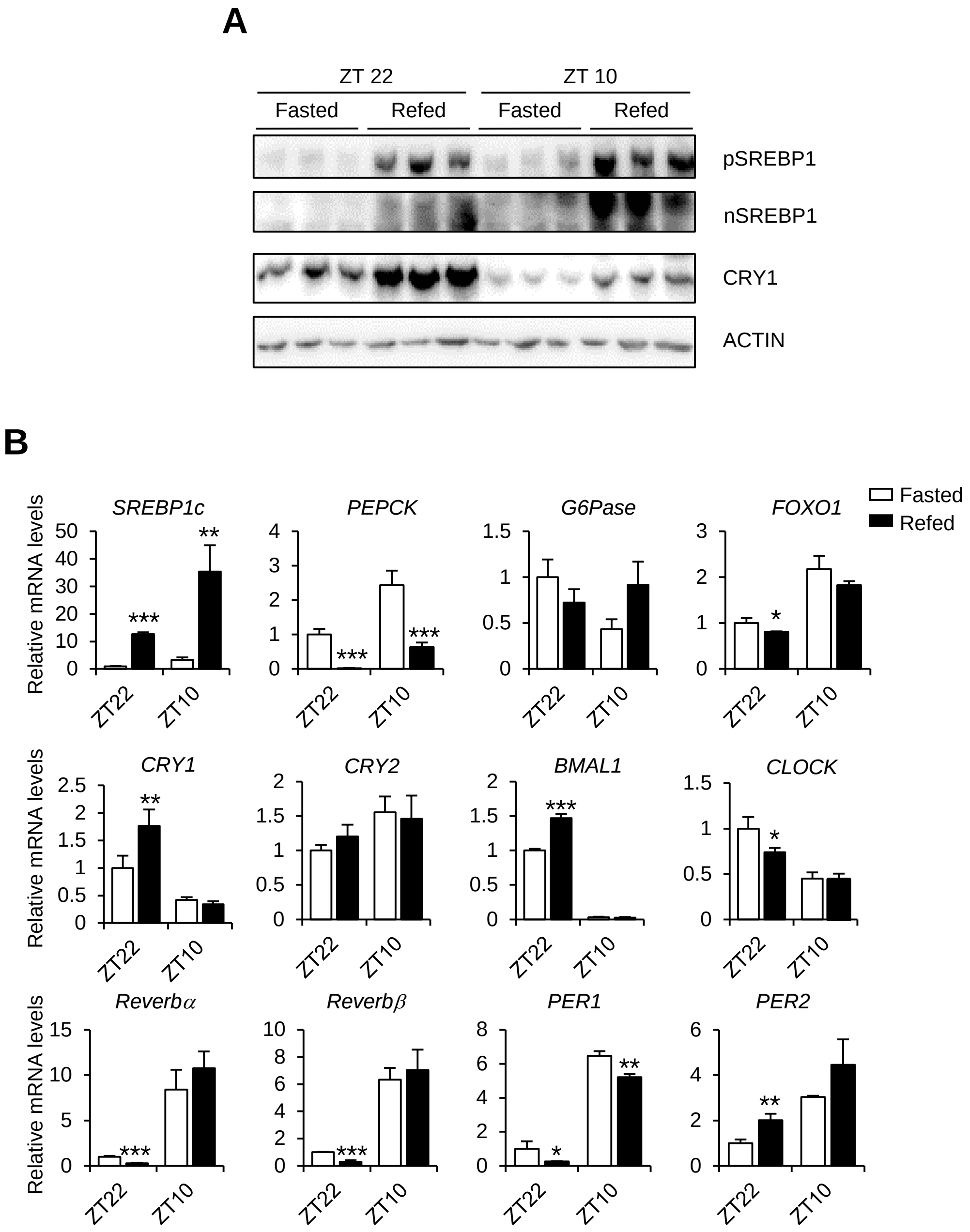

Supplementary Figure 2. CRY1 is increased by feeding regardless of time periods

(A and B) *C57BL/6* mice were fasted for 24 hours and then refed for 12 hours. Both fasted and refed mice were sacrificed at ZT 22 and ZT 10. In the liver, the levels of CRY1 protein (A) and *CRY1* mRNA (B) were determined using western blotting and qRT-PCR with normalization to *TBP* mRNA levels, respectively. Data are represented as mean ±SD, N=3 for each group. *$P<0.05$, **$P<0.01$, ***$P<0.001$ (Student's *t*-test).

Supplementary Figure 3

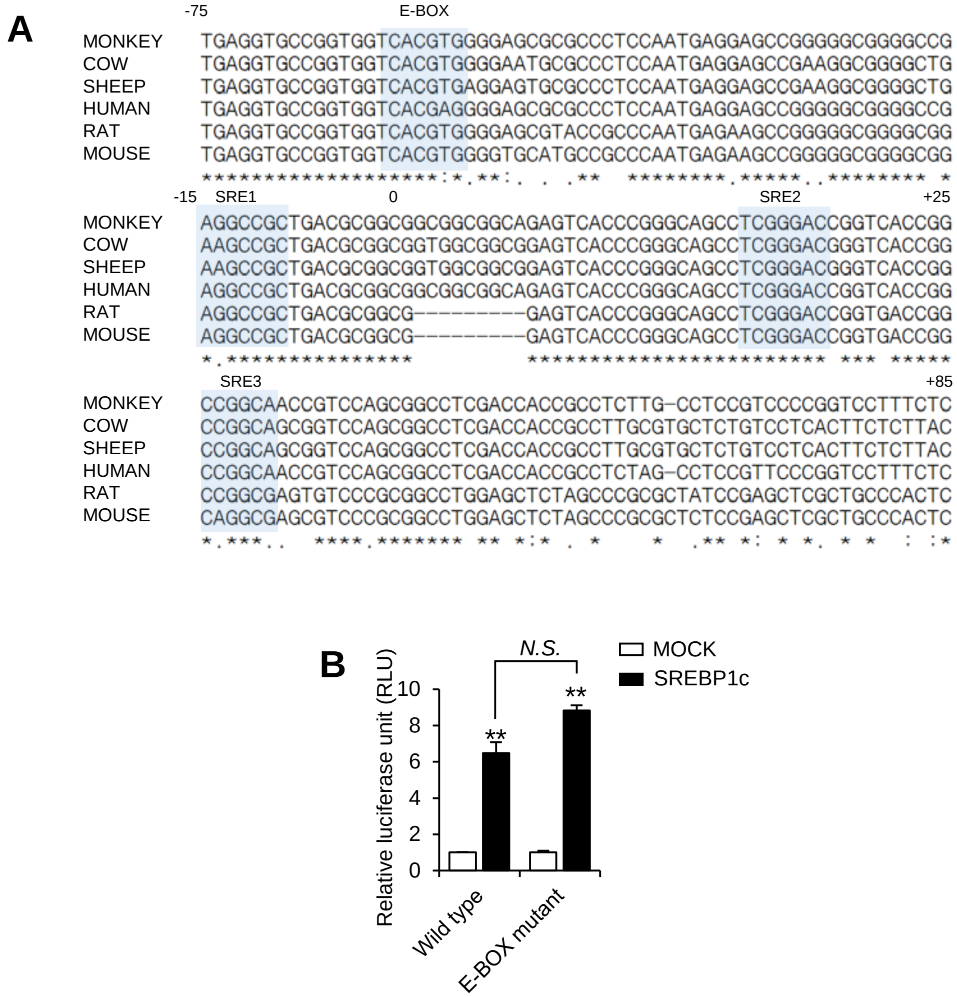

Supplementary Figure 3. CRY1 promoter analysis using luciferase assay

(A) SRE motifs and E-BOX sequences in the CRY1 promoter from various species. (B) HEK293T cells were co-transfected with a reporter plasmid containing the WT or E-BOX mutant mouse CRY1 promoter along with expression plasmids encoding either MOCK or SREBP1c. The values represent the mean ± SD (N=4 for each group). *P<0.05, **P<0.01 (Student’s *t*-test).

Supplementary Figure 4

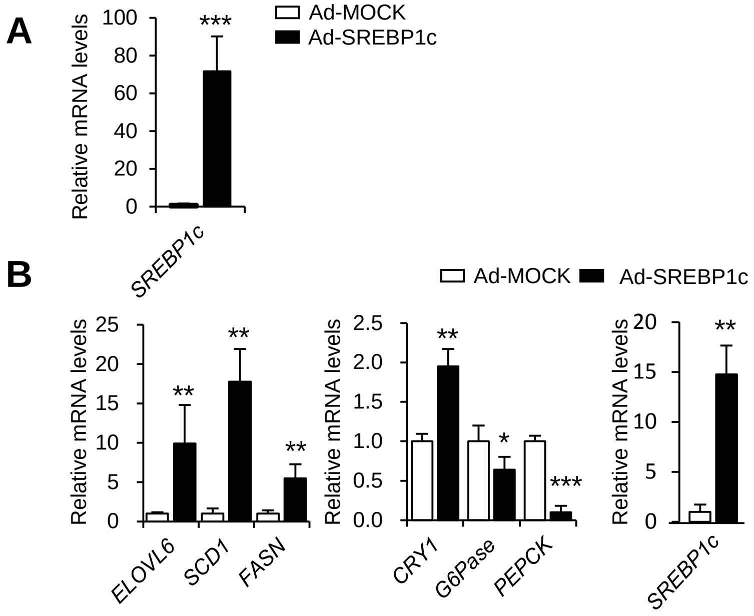

Supplementary Figure 4. Gene expression profiles

(A) Mouse primary hepatocytes were adenovirally infected with Ad-MOCK or Ad-SREBP1c in Figure 2A and 2B. The levels of the *SREBP1c* mRNA were determined using qRT-PCR with normalization to *TBP* mRNA levels. Data are represented as mean ±SD, *N*=3 for each group. *$P<0.05$, **$P<0.01$, ***$P<0.001$ (Student's *t*-test). (B) *C57BL/6* mice were infected with adenoviruses encoding either MOCK or SREBP1c (adenoviral dose of 5×10^9 viral particles per mouse) through the tail vein in Figure 3B. Mice were sacrificed 5 days following the injection of adenoviruses and relative mRNA levels were determined by qRT-PCR and normalized to the *TBP* mRNA levels. Data are represented as mean ±SD, *N*=3-4 for each group. *$P<0.05$, **$P<0.01$, ***$P<0.001$ (Student's *t*-test).

Supplementary Figure 5

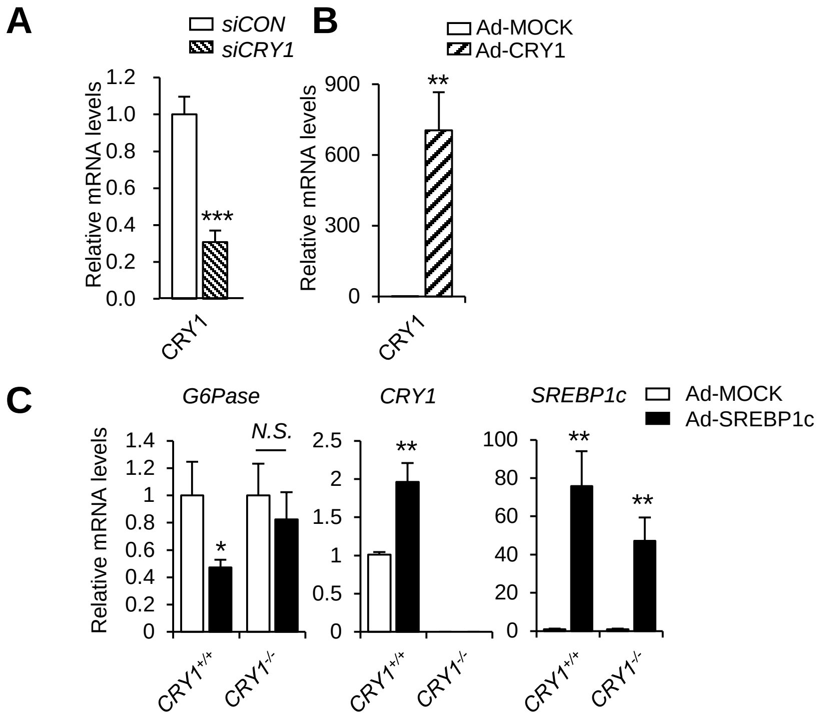

Supplementary Figure 5. Gene expression profiles

(A) H4IIE cells were transfected with siCON or siCRY1 as shown in Figure 3G. Relative *CRY1* mRNA levels were determined by qRT-PCR and normalized to *cyclophilin* mRNA levels. Data are represented as mean ±SD, *N*=3 for each group. ****P<0*.001 (Student's *t*-test). (B) Mouse primary hepatocytes were infected with Ad-MOCK or Ad-CRY1 in Figure 3H. Relative *CRY1* mRNA levels were determined by qRT-PCR and normalized to *TBP* mRNA levels. Data are represented as mean ±SD, *N*=3 for each group. ***P<0*.01 (Student's *t*-test). (C) Mouse primary hepatocytes isolated from *CRY1*$^{+/+}$ and *CRY1*$^{-/-}$ mice were infected with Ad-MOCK or Ad-SREBP1c in Figure 3K. Relative mRNA levels were determined by qRT-PCR and normalized to *TBP* mRNA levels. Data are represented as mean ±SD, *N*=3 for each group. ***P<0*.01 (Student's *t*-test).

Supplementary Figure 6

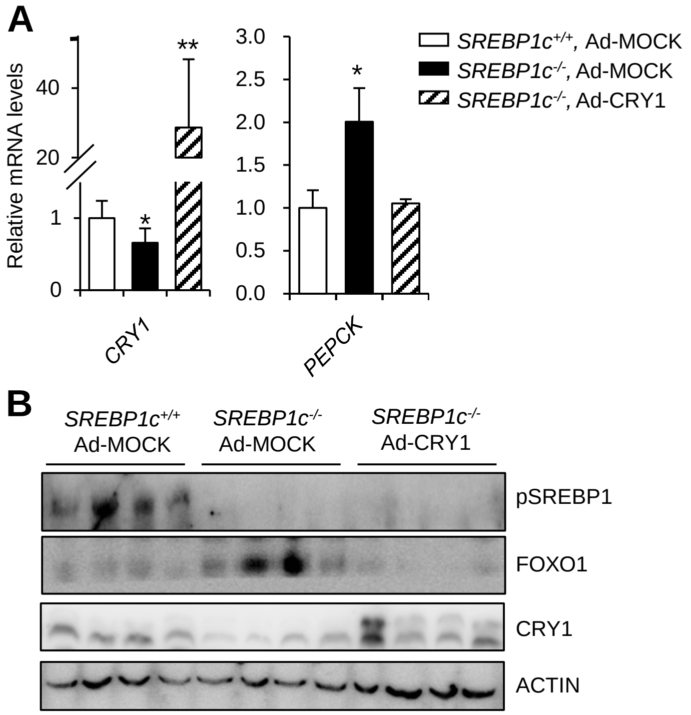

Supplementary Figure 6. CRY1 overexpression reduces *PEPCK* mRNA and FOXO1 protein levels in *SREBP1c*$^{-/-}$ mice

(A and B) *SREBP1c*$^{+/+}$ mice injected with Ad-MOCK and in *SREBP1c*$^{-/-}$ mice injected with either Ad-MOCK or Ad-CRY1 in Figure 3L and 3M. In the liver, levels of the mRNA (A) and protein (B) were determined using qRT-PCR with normalization to *TBP* mRNA levels and western blotting, respectively. Data are represented as mean ±SD, *N*=7~10 for each group. *$P<0.05$, **$P<0.01$ (Student's *t*-test).

Supplementary Figure 7

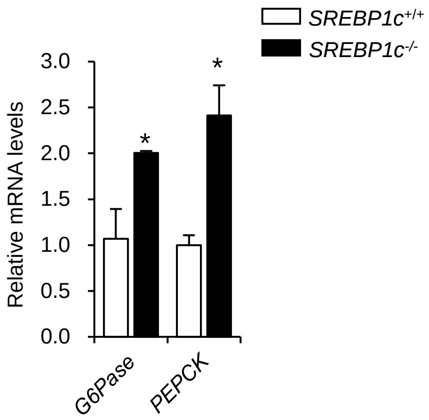

Supplementary Figure 7. Gluconeogenic gene expression is elevated in *SREBP1c*$^{-/-}$ mice
In the liver, the levels of the *G6Pase* and *PEPCK* mRNA were determined using qRT-PCR with normalization to *TBP* mRNA levels. Data are represented as mean ±SD, *N*=3~4 for each group. *$P<0.05$ (Student's *t*-test).

Supplementary Figure 8

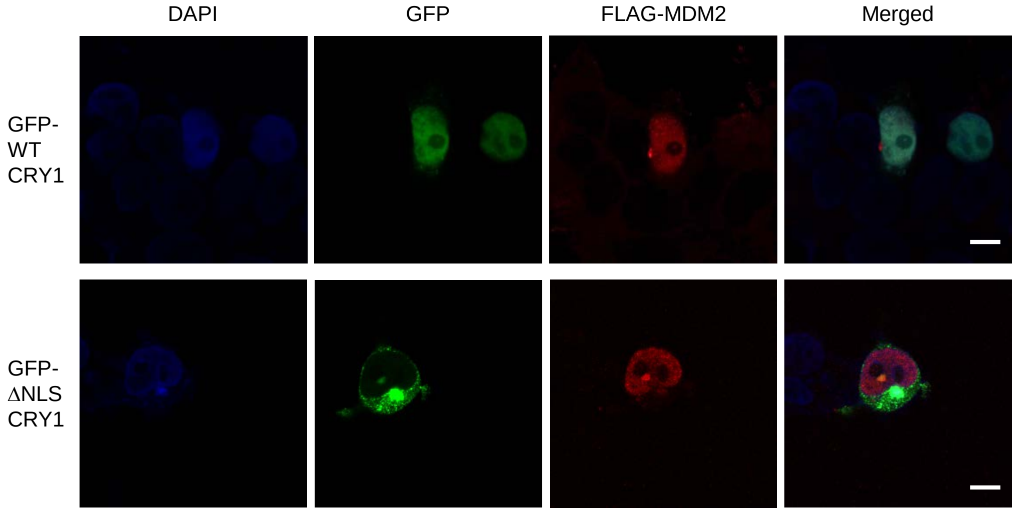

Supplementary Figure 8. MDM2 subcellular localization in WT CRY1 and cytosolic CRY1 overexpressing cells

FLAG-MDM2 was co-transfected with GFP-WT CRY1 or GFP-cytosolic CRY1 (ΔNLS CRY1) in HEK293T cells. Immunocytochemical analysis of FLAG-MDM2 and GFP was carried out. DAPI, 4',6-diamidino-2-phenylindole. Scale bars=10 μm.

Supplementary Figure 9

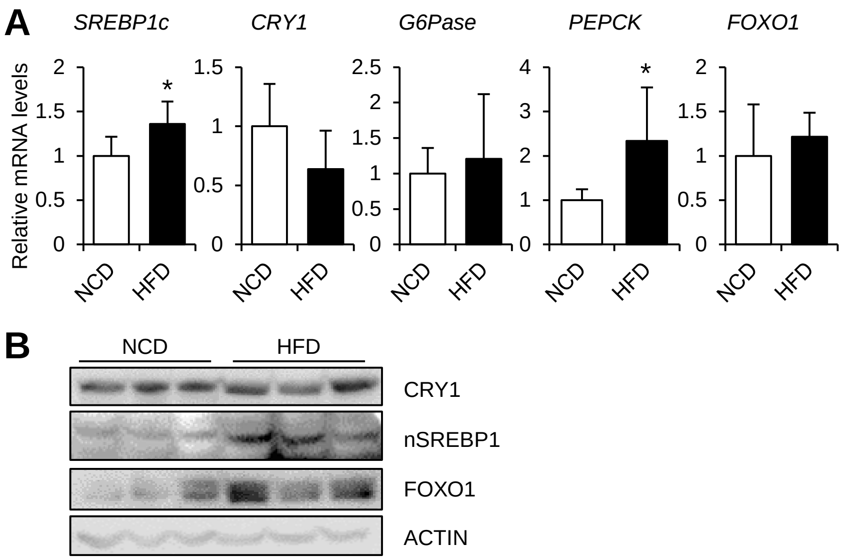

Supplementary Figure 9. Expression profiles of various genes in the liver of NCD and HFD fed mice

(A and B) Eight-week-old *C57BL/6* mice were fed a NCD or HFD for 8 weeks and sacrificed at ZT3. Hepatic gene expression levels (A) were determined by qRT-PCR and normalized by the level of the *TBP* mRNA. N=5 in each group. Hepatic protein levels (B) were analyzed by western blotting.

Supplementary Figure 10

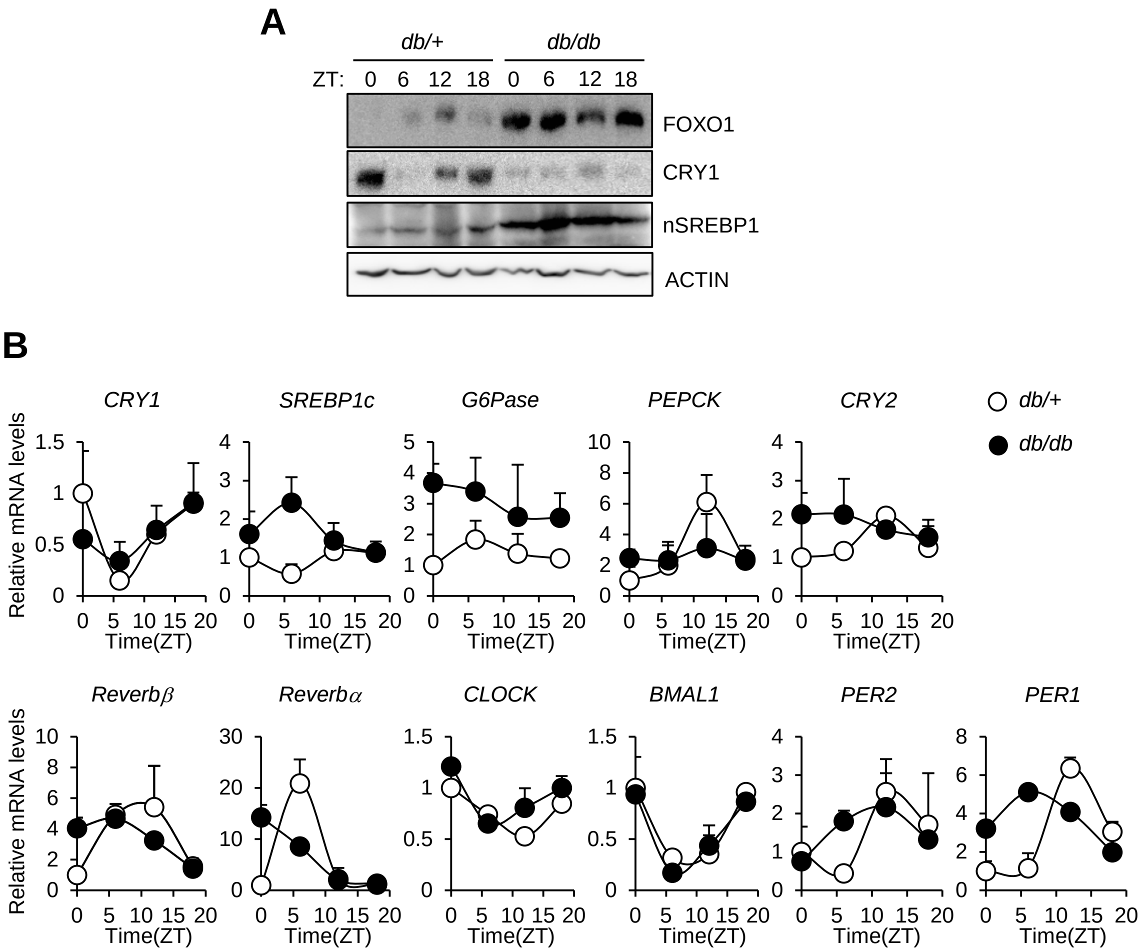

Supplementary Figure 10. Diurnal gene expression profiles of *db/+* and *db/db* mice liver

(A and B) Ten-week-old male *db/+* and *db/db* mice were sacrificed at ZT 0, 6, 12, and 18. Protein levels (A) were determined with western blotting. The relative mRNA levels of various hepatic genes (B) were determined by qRT-PCR analyses and normalized to the *TBP* mRNA level. Data are represented as mean ±SD, *N*=2 for each group.

Supplementary Figure 11

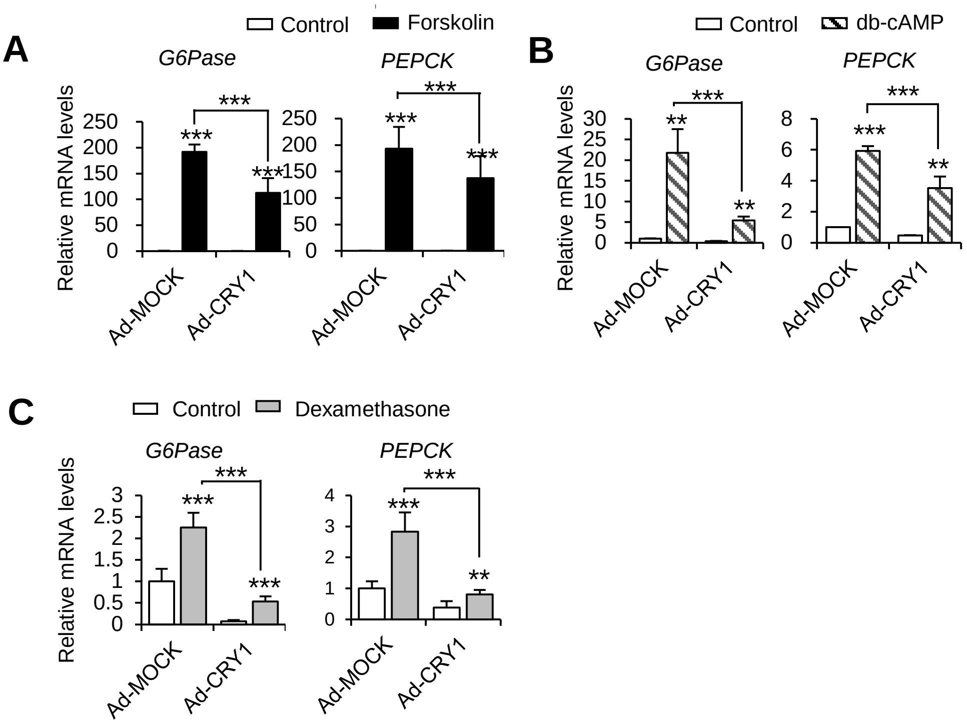

Supplementary Figure 11. Gluconeogenic gene expression is partially attenuated by CRY1 overexpression upon incubation with forskolin, db-cAMP, and dexamethasone

(A, B and C) Mouse primary hepatocytes were adenovirally infected with Ad-MOCK or Ad-CRY1. The cells were treated with 10 µM forskolin (A), 5 µM db-cAMP (B), and 1 µM dexamethasone (C) or vehicle (Control) for 4 hours. *PEPCK* and *G6Pase* mRNA levels were determined using qRT-PCR and normalized to the level of the *TBP* mRNA. The values represent the mean ± SD (N=3 for each group). *P<0.05, **P<0.01, ***P<0.001 (Student's *t*-test).

Supplementary Figure 12

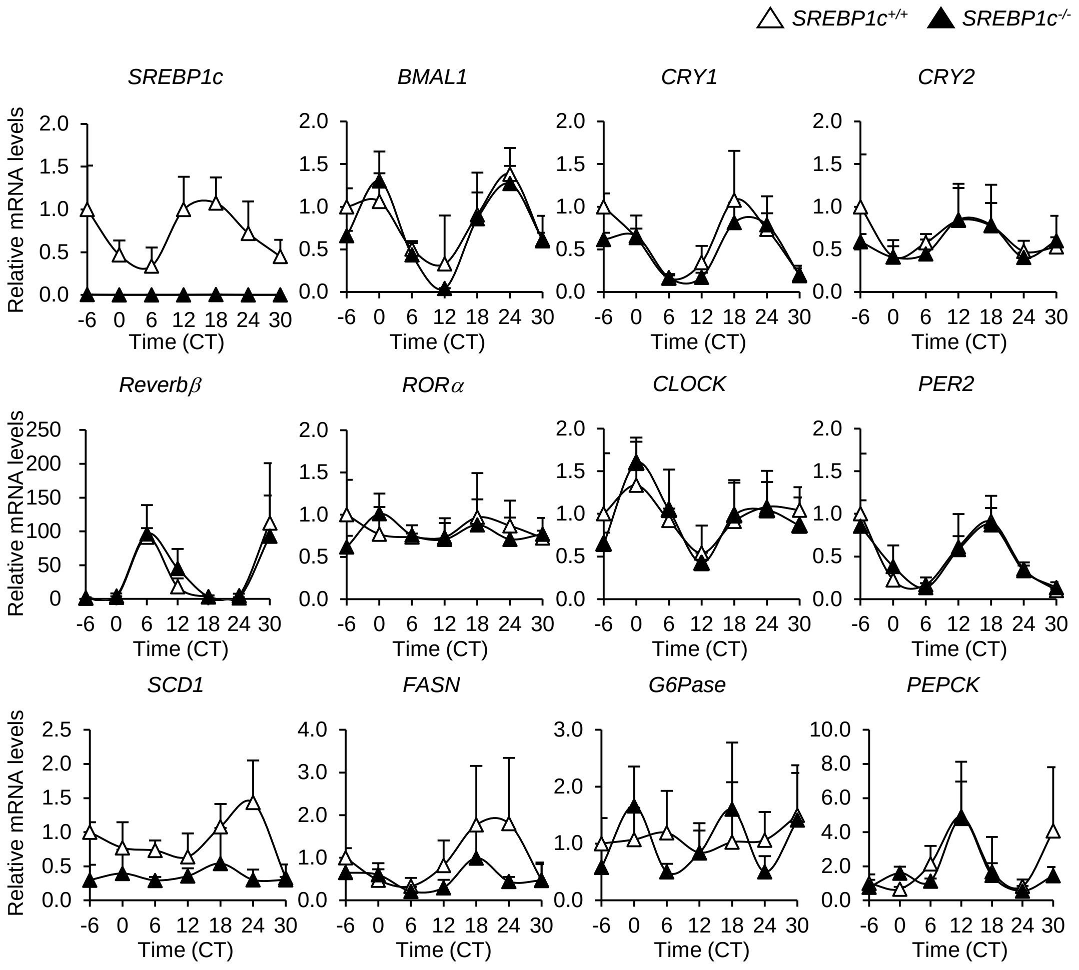

Supplementary Figure 12. Expression profiles of various genes in the liver of *SREBP1c*$^{+/+}$ and *SREBP1c*$^{-/-}$ mice

Livers were isolated every 6 hours from *SREBP1c*$^{+/+}$ and *SREBP1c*$^{-/-}$ mice fed with normal chow diet and kept under 12 hours: dark, 12 hours: dark cycle for 1 week. Relative mRNA levels were determined by qRT-PCR and normalized by the level of the *TBP* mRNA. N=3-4 at each time point.

Supplementary Figure 13

Figure 1B

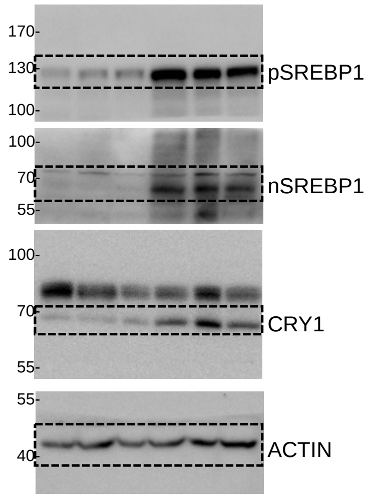

Figure 2B

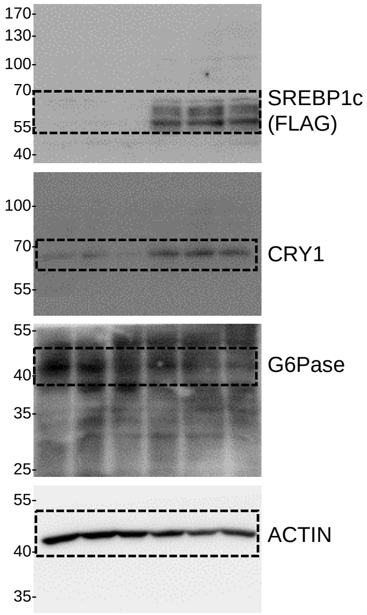

Figure 2E

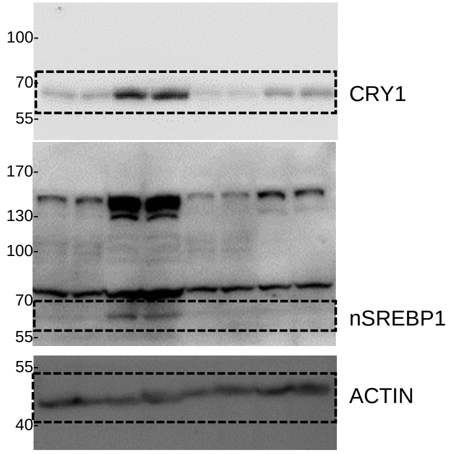

Figure 4A

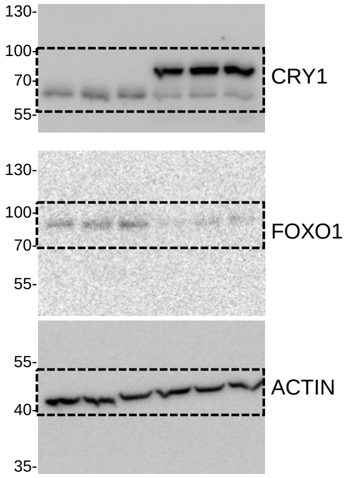

Figure 4D

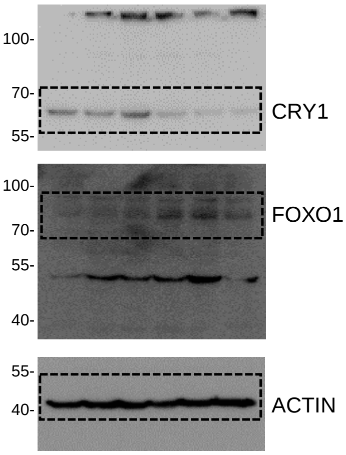

Figure 4E

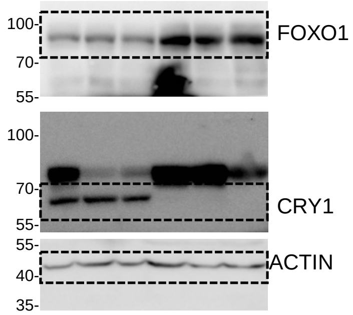

Figure 4G

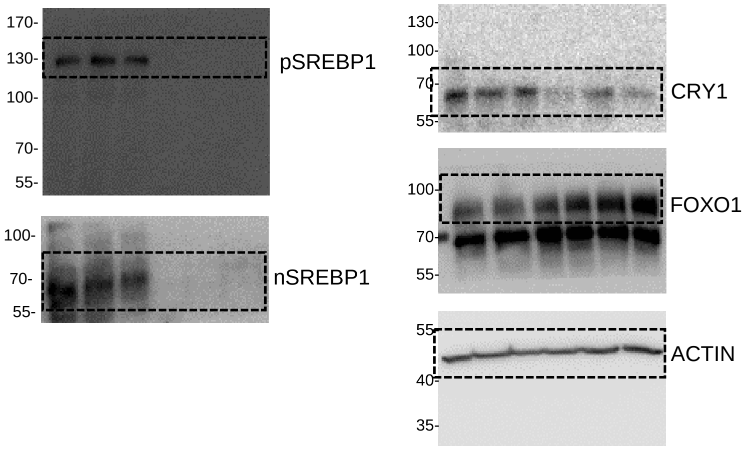

Figure 5A

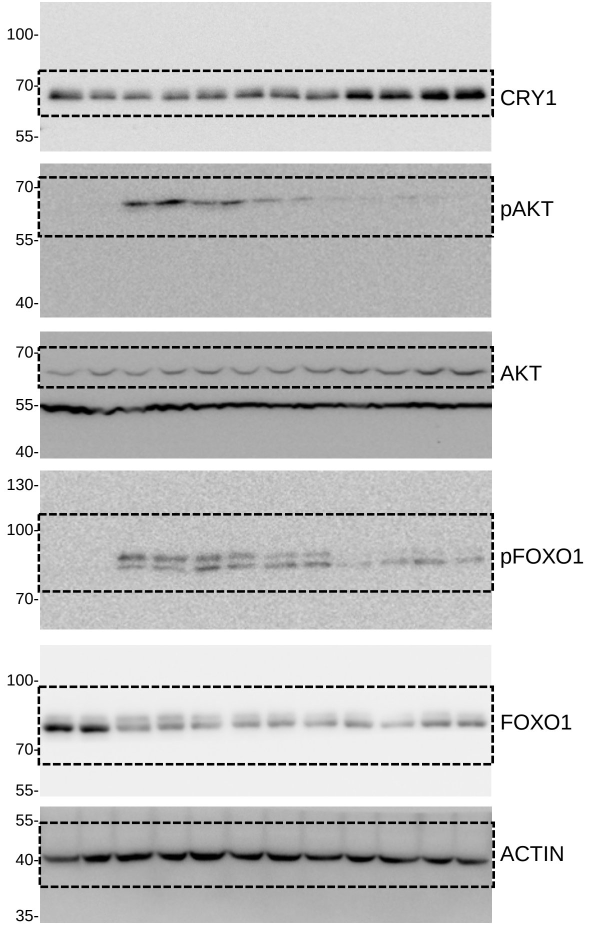

Figure 5C

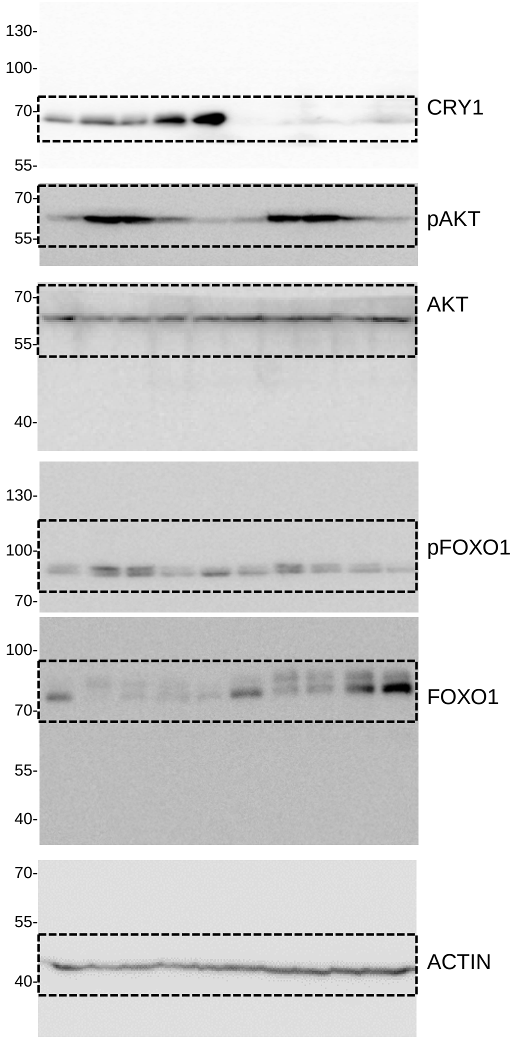

Figure 5F

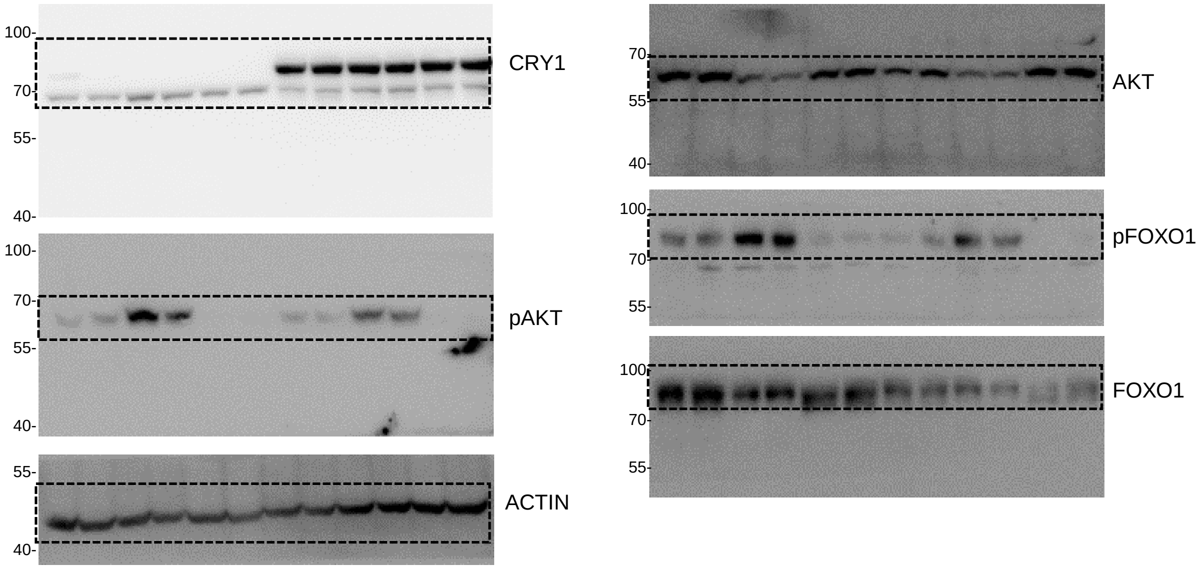

Figure 5I

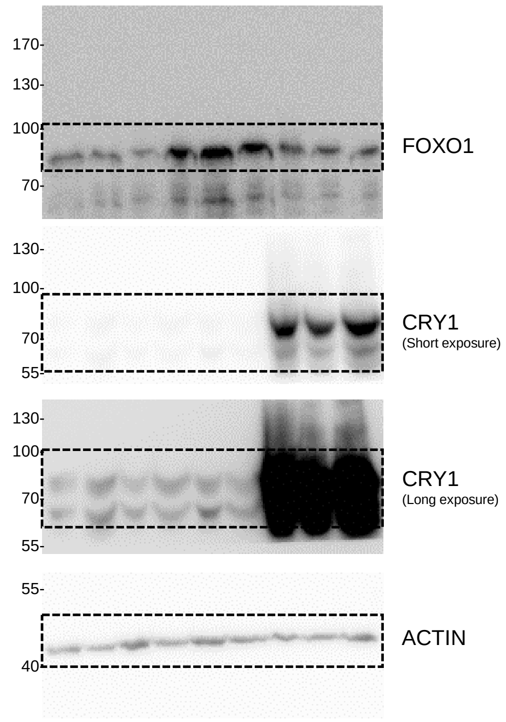

Figure 6A

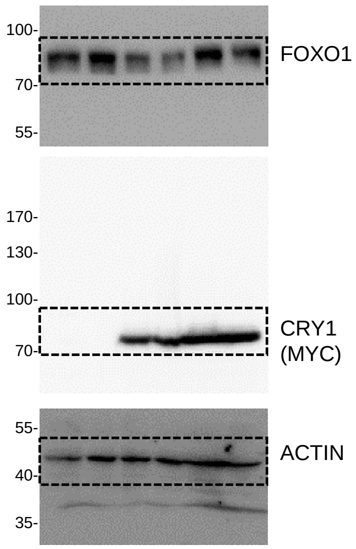

Figure 6B

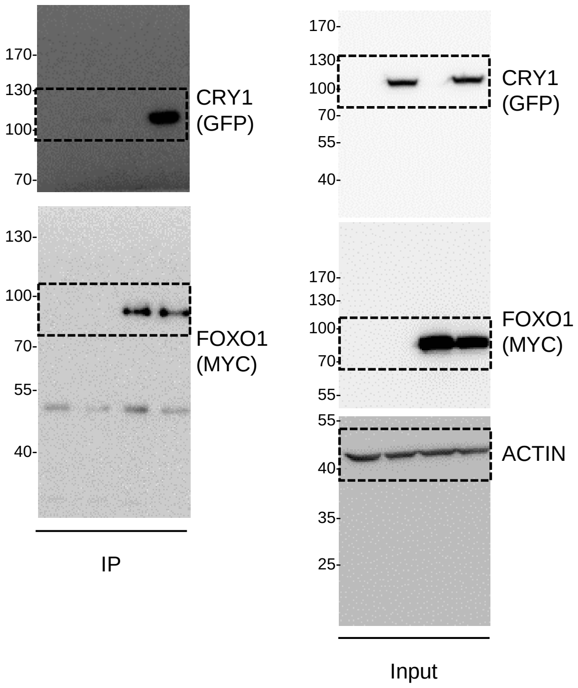

Figure 6C

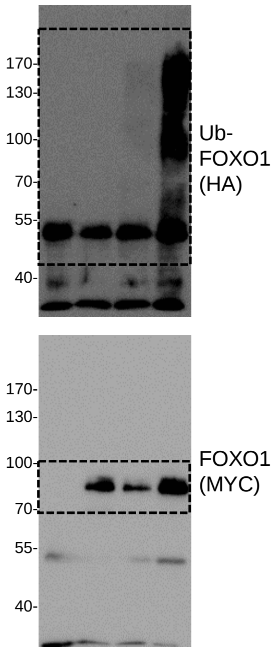

Figure 6D

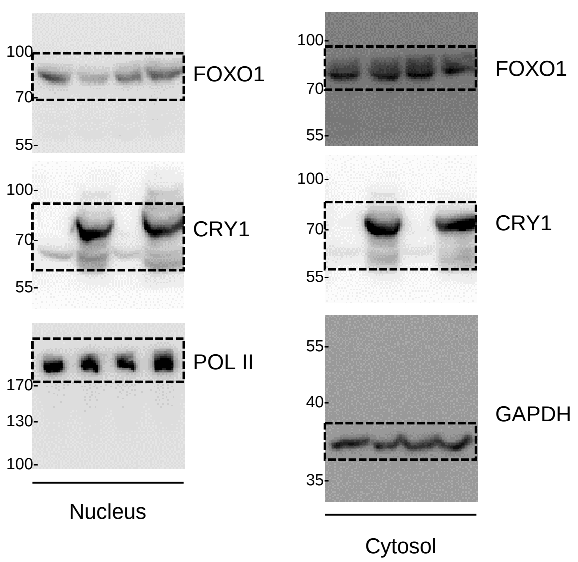

Figure 6E

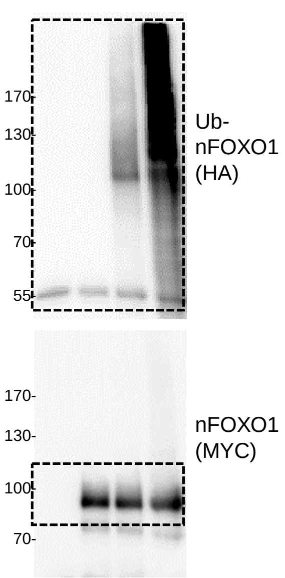

Figure 7A

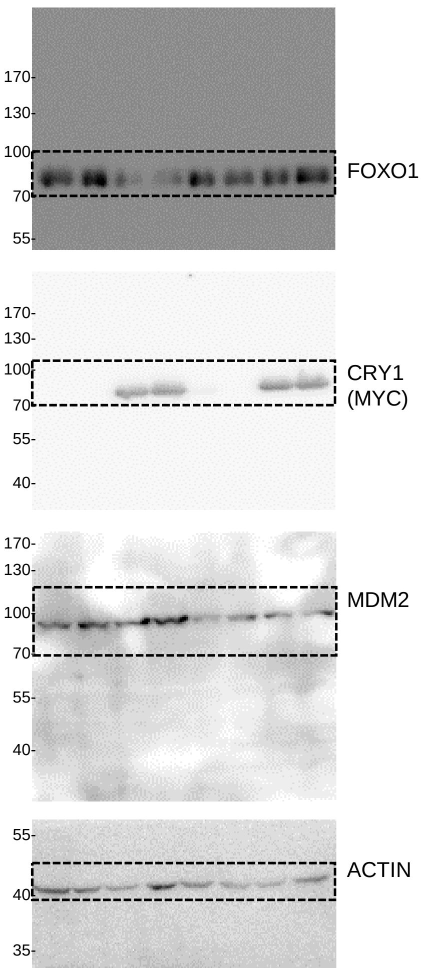

Figure 7B

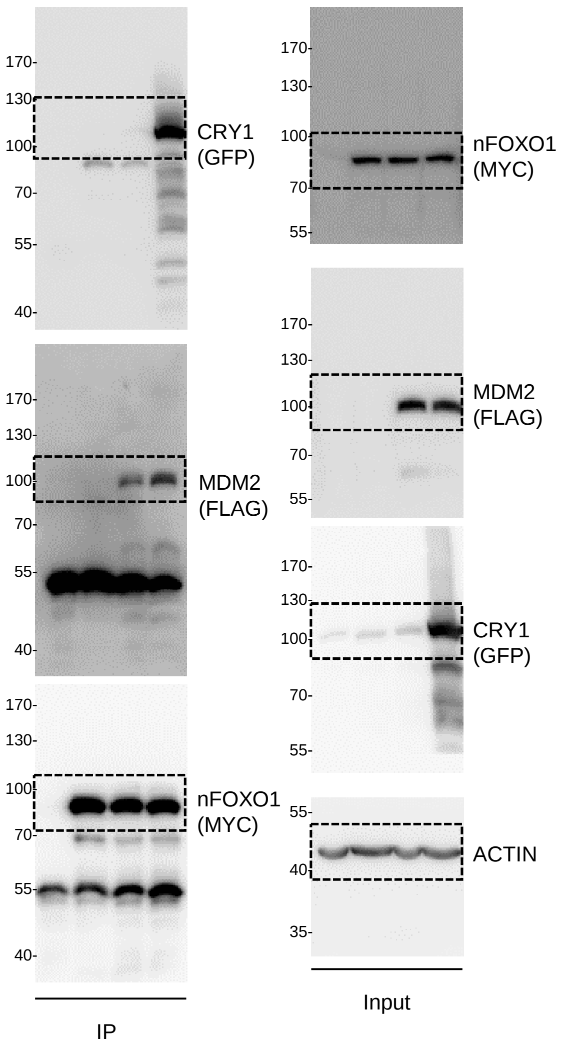

Figure 7C

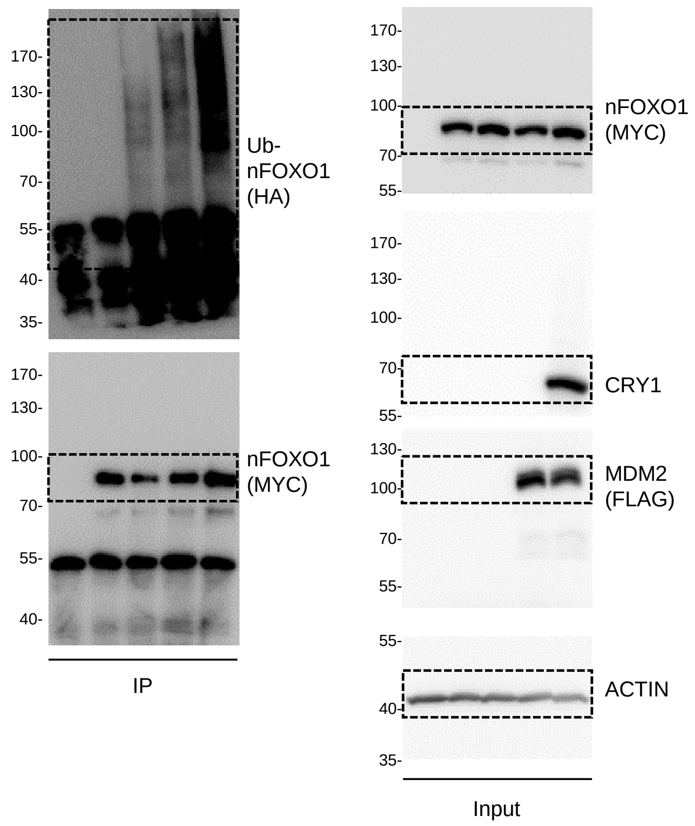

Figure 7C

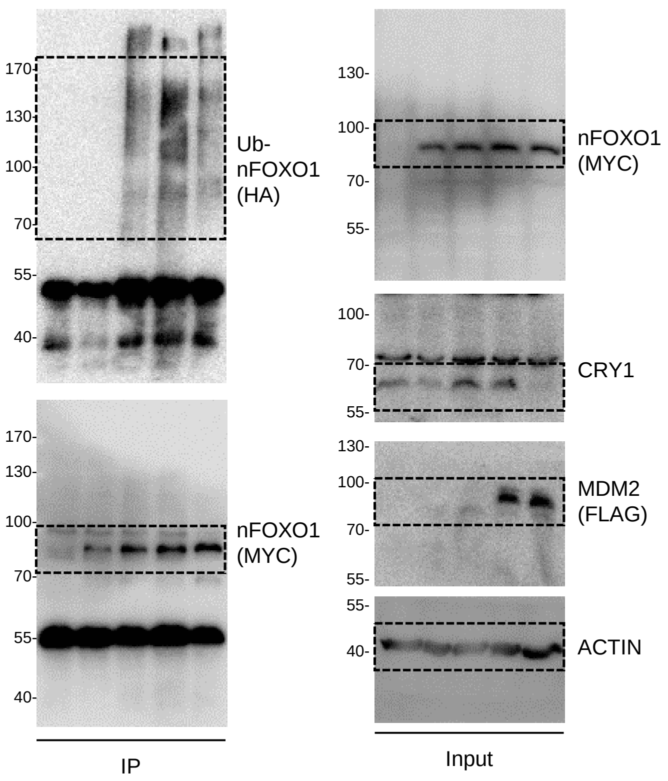

Figure 8B

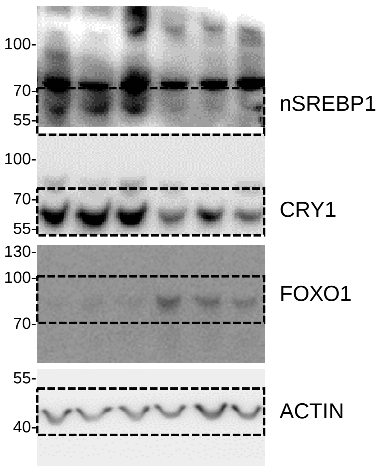

Figure 8F

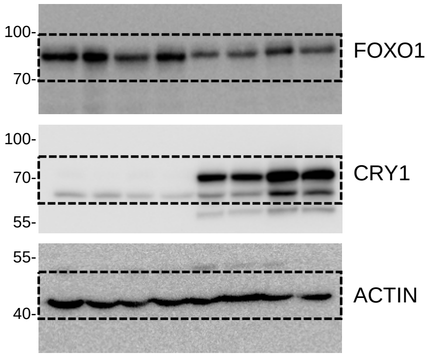

Figure 8H

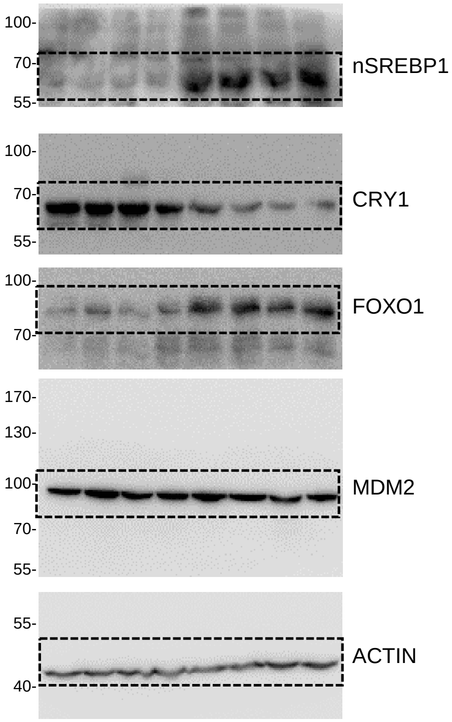

Figure 8K

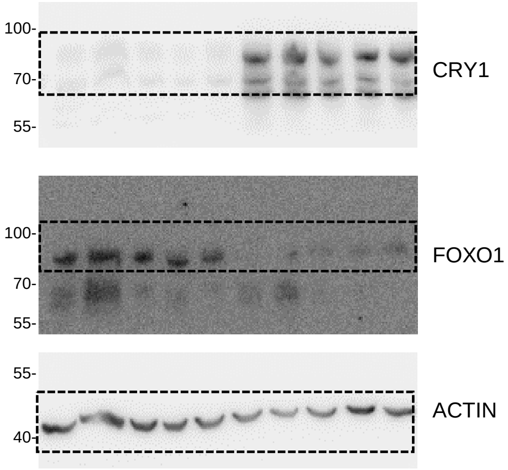

Supplementary figure 1B

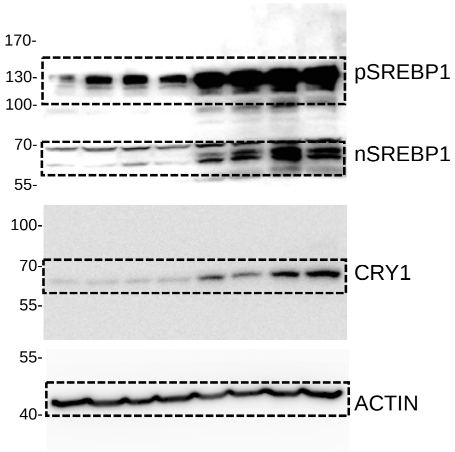

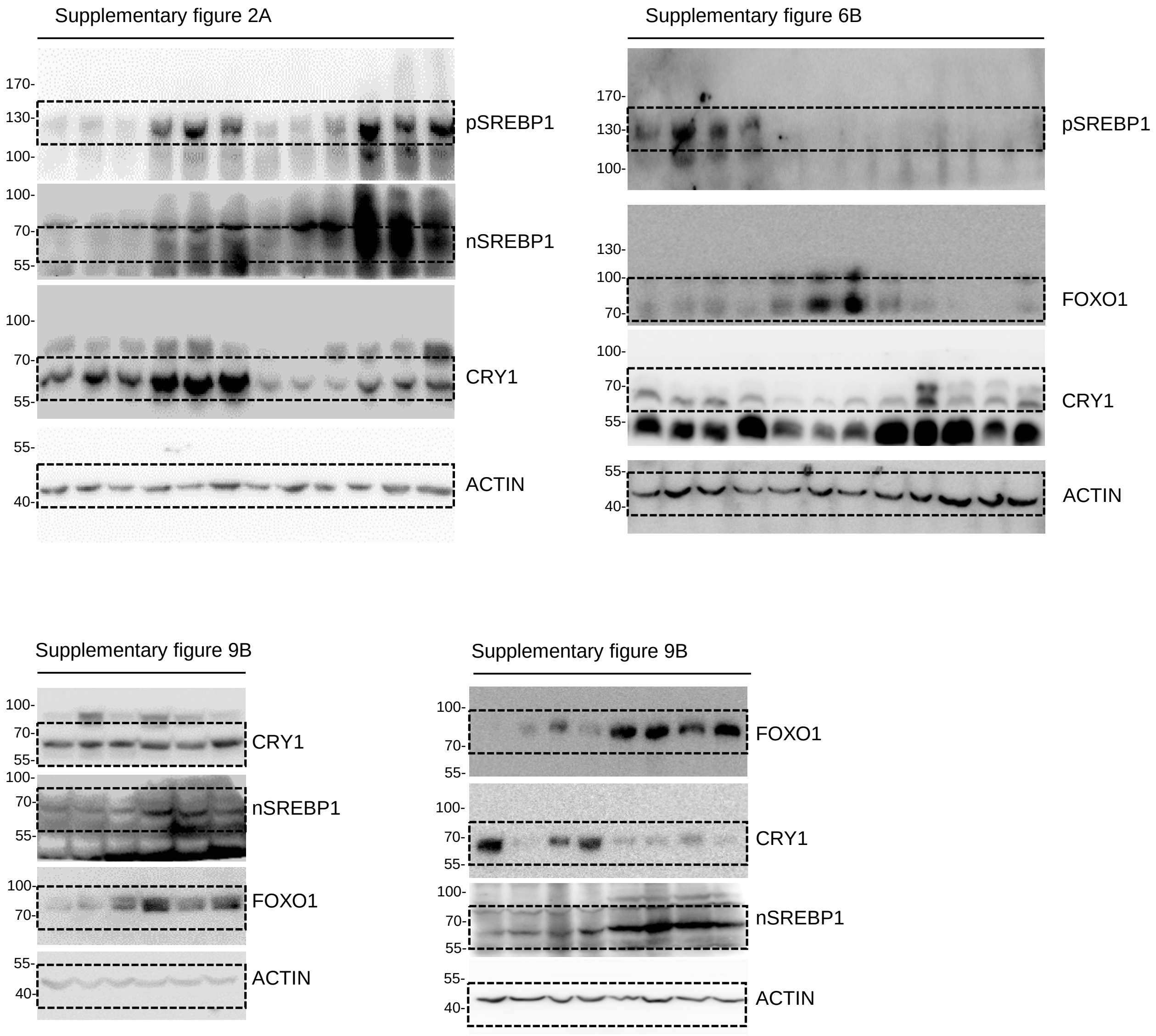

Supplementary Figure 13. The original full immunoblot presented in main figures and supplementary figures

Supplementary Table 1. Sequence of primers for q-RT-PCR

Primer	Sequences
mrSREBP1c	Sense 5'-GGAGCCATGGATTGCACATT<br>Antisense 5'-CAGGAAGGCTTCCAGAGAGG
mrCRY1	Sense 5'-CGTTTGGAAAGGCATTTGG<br>Antisense 5'-CTTCCATTTTGTCAAAGCGTG
mrPEPCK	Sense 5'-AGCCTTTGGTCAACAACTGG<br>Antisense 5'-TGCCTTCGGGGTTAGTTATG
mrG6Pase	Sense 5'-ACACCGACTACTACAGCAACAG<br>Antisense 5'-CCTCGAAAGATAGCAAGAGTAG
hmrFOXO1	Sense 5'-CCAAGGCCATCGAGAGC<br>Antisense 5'-GATTGAGCATCCACCAAGAACT
hmFASN	Sense 5'-GCTGCGGAAACTTCAGGAAAT<br>Antisense 5'-AGAGACGTGTCACTCCTGGACTT
mBMAL1	Sense 5'-AACCTTCCCGCAGCTAACAG<br>Antisense 5'-AGTCCTCTTTGGGCCACCTT
mCLOCK	Sense 5'-TTGCGTCTGTGGGTGTTG<br>Antisense 5'-TGCTTTGTCCTTGTCATCTTCT
mPER2	Sense 5'-TGTGCGATGATGATTCGTGA<br>Antisense 5'-GGTGAAGGTACGTTTGGTTTGC
hmrCRY2	Sense 5'-GGAGCTGCCCAAGAAGC<br>Antisense 5'-AGTAGAAGAGGCGGCAGGA
mSCD1	Sense 5'-CCGGAGACCCCTTAGATCGA<br>Antisense 5'-TAGCCTGTAAAGATTTCTGCAAACC
mELOVL6	Sense 5'-TGCCATGTTCATCACCTTGT<br>Antisense 5'-TACTCAGCCTTCGTGGCTTT
mTBP	Sense 5'-GGGAGAATCATGGACCAGAA<br>Antisense 5'-CCGTAAGGCATCATTGGACT
mPER1	Sense 5'-GGGAGCTCAAACTTCGACTG<br>Antisense 5'-TCGGATGTGATATGCTCCAA
mREVERBα	Sense 5'-CTGGAGGGCTGCAGTATAGC<br>Antisense 5'-TATTGGAGTCCAGGGTCGTC
mREVERBβ	Sense 5'-TCCTCTAGCTCTGCCTCCAG<br>Antisense 5'-TGGTTTTGCCTGTTTTCACA

(m: mouse, h: human, r: rat)

Supplementary Table 2. siRNA Sequences

Primer	Sequences
Rat FOXO1	5’-GAAUGAAGGAACUGGGAAA
Mouse CRY1	5’-CCUCGCAACUGAAGUUGGU
Mouse MDM2	5’-CAGAGAAUGAUGGUAAAGA
Negative Control	5’-CCUACGCCACCAAUUUCGU
